# Enhancing Ferroptosis in Lung Adenocarcinoma Cells via the Synergistic Action of Nonthermal Biocompatible Plasma and a Bioactive Phenolic Compound

**DOI:** 10.3390/biom15050691

**Published:** 2025-05-09

**Authors:** Sabnaj Khanam, Young June Hong, Youngsun Kim, Eun Ha Choi, Ihn Han

**Affiliations:** 1 Department of Plasma Bio-Display, Kwangwoon University, Seoul 01897, Republic of Korea; sabnajkhanam719@gmail.com; 2 Plasma Bioscience Research Center (PBRC), Kwangwoon University, Seoul 01897, Republic of Korea; cosm98@gmail.com; 3 Advanced Technology Research Institute, Nayuda Co., Seoul 04067, Republic of Korea; 4 Department of Obstetrics and Gynecology, Kyung Hee University Medical Center, Seoul 02447, Republic of Korea; chacha0725@naver.com; 5 Department of Electronic and Biological Physics, Kwangwoon University, Seoul 01897, Republic of Korea

**Keywords:** para-coumaric acid (p-CA), nonthermal biocompatible pressure plasma (NBP), ferroptosis, lung adenocarcinoma cells, co-treatment

## Abstract

Para-coumaric acid (p-CA) is a phenolic compound that has antioxidant, anti-inflammatory, and anticancer properties which make it potential for cancer treatment. However, its effectiveness has been limited by poor solubility, rapid metabolism, and poor absorptivity. Nonthermal biocompatible pressure plasma (NBP) has gained attention as a cancer treatment due to its ability to generate reactive oxygen and nitrogen species (RONS), inducing oxidative stress that damages cancer cells. This study aimed to investigate the combined effect of NBP and p-CA on the induction of ferroptosis in lung adenocarcinoma via the GPX4, xCT, and NRF2 pathways. H460 and A549 lung adenocarcinoma cells as well as normal lung cells (MRC5) were treated with p-CA, NBP, and their combination. Cell movement, intracellular RONS levels, and lipid peroxidation, along with apoptosis and ferroptosis-related gene expression, were evaluated by co-treatment. Co-treatment also significantly elevated NO_2_^−^, NO_3_^−^, and H_2_O_2_ levels and reduced cancer cell (H460, A549) viability (26, 31%) without affecting normal cells MRC5 (7%). Elevated MDA levels and changed expression of ferroptotic proteins indicated mitochondrial dysfunction, oxidative damage, lipid peroxidation, and DNA damage, which resulted in the induction of ferroptosis. These findings reveal a novel ferroptosis mechanism, emphasizing co-treatment for delivering bioavailable natural anticancer drugs.

## 1. Introduction

Lung cancer is the leading cause of cancer death worldwide [1], especially non-small cell lung cancer (NSCLC), which is responsible for most cases and 25% of cancer-related deaths. In addition, surgery, chemotherapy, and radiation therapy are standard treatments for lung adenocarcinoma. Similarly, immunotherapy and targeted medicines are also new possibilities for curing these diseases [2]. However, patients with lung adenocarcinoma have poor survival rates due to increased drug resistance and recurrence [3]. Therefore, there is an urgent need to explore new therapeutic targets and innovative approaches to improve lung cancer treatment.

Programmed cell death (PCD), a regulated process controlled by certain genes and molecular pathways, plays an essential role in the normal growth and homeostasis of multicellular organisms [4]. Among the most well-known forms of PCD are ferroptosis, pyroptosis, necroptosis, autophagy, and apoptosis [5]. Ferroptosis, a recently identified type of programmed cell death, is characterized by iron dependency and lipid peroxidation accumulation, which disrupts the cellular redox balance and ultimately results in cell death [4]. It causes mitochondrial shrinkage, fewer cristae, and denser membranes [2]. Notably, ferroptosis-based cancer therapy offers a novel approach to combat chemotherapy resistance by inducing apoptosis through nonapoptotic pathways. Additionally, xCT is a potent membrane antiporter that stimulates GSH synthesis, maintains redox balance, protects cells from ferroptosis, and exchanges internal glutamate for extracellular cystine [6]. Several studies have reported that regulators such as mTORC1, AMPK, p53, and BAP1 play crucial roles in modulating xCT expression and controlling ferroptosis [7]. p53 also promotes ferroptosis by inhibiting xCT expression via the p53-SLC7A11 axis, increasing ROS levels, and blocking cystine uptake, demonstrating the close connection between ferroptosis and apoptosis [8,9,10]. Diseases, including ischemia–reperfusion injury, renal failure, and neurodegeneration, can be caused by excessive ferroptosis, whereas cancer progression is associated with insufficient ferroptosis [11]. Currently, ferroptosis resistance and sensitivity are subjected to cancer therapy. Therefore, alternative cancer treatments that are affordable, safe, and effective need to be identified.

Nonthermal biocompatible plasma (NBP) is an ionized gas containing reactive species, including electrons, ions, neutral molecules, and ultraviolet photons [12,13]. Recent studies have shown that NBP has significant anticancer effects both in vitro and in vivo [14,15], suggesting its possible use in cancer therapy. Mechanistically, NBP elevates the levels of intracellular reactive oxygen–nitrogen species (RONS), induces oxidative stress, damages macromolecules, and ultimately triggers cell death [16]. Numerous studies have demonstrated that NBP damages DNA by generating reactive oxygen–nitrogen species (RONS) and impairs mitochondrial function by promoting the accumulation of intracellular chelatable iron [16,17]. Furthermore, NBP has been shown to trigger a variety of programmed cell death processes, such as apoptosis [18], autophagy [19], pyroptosis [20], and senescence [21], which are gaining increasing recognition in the field of oncology.

On the other hand, para-coumaric acid (p-CA), a hydroxy derivative of cinnamic acid found in onion, carrot, and tomato, has multiple pharmacological properties, including antioxidant, antimicrobial, and anticancer activities [22]. It also induces apoptosis and necrosis, inhibits angiogenesis, and reduces inflammation in cancer cells [23]. Epidemiological studies have suggested that p-CA can inhibit diseases by scavenging free radicals and increasing cardiovascular and immune functions [24]. However, the therapeutic potential of p-CA is often limited because of its low bioavailability and biocompatibility [25,26]. To overcome these limitations, the combination of NBP and p-CA could be an effective strategy.

NBP induces oxidative stress by producing reactive oxygen and nitrogen species (RONS), whereas p-CA increases the vulnerability of cancer cells to lipid peroxidation. Co-treatment can disrupt the cellular redox balance, promote lipid peroxidation, and increase the level of intracellular iron, leading to ferroptosis. In addition to increasing iron availability, NBP catalyzes the Fenton reaction, which results in hydroxyl radicals that accelerate oxidative damage [27]. Additionally, co-treatment impairs the antioxidant defenses of cancer cells, decreasing their resistance to ferroptosis. By taking advantage of their distinct redox imbalance and iron metabolism [28] without damaging normal cells, co-treatment triggers lung adenocarcinoma cell death. By enhancing the solubility, stability, and targeted delivery of p-CA, NBP overcomes pharmacokinetic challenges and enhances its anticancer effects, suggesting a promising direction for future cancer therapies.

This study aimed to establish a novel therapeutic approach by combining the selective killing properties of NBP and the anticancer potential of para-coumaric acid. Co-treatment effectively inhibited the migratory behavior of lung adenocarcinoma cells. Additionally, the study examined the expression of the key proteins involved in the apoptosis and ferroptosis pathways. Furthermore, NBP with p-CA triggers lipid peroxidation and increases chelatable iron levels, indicating alterations in the amounts of proteins involved in ferroptosis. By investigating the molecular mechanisms behind this combined therapy, the research aims to contribute to the advancement of more selective and effective targeted cancer treatments.

## 2. Materials and Methods

### 2.1. Reagents

Para-coumaric acid (C9008, Sigma Aldrich, Seoul, Republic of Korea) and phosphate-buffered saline (PBS) (LB 001–02, Welgene, Gyeongsan-si, Republic of Korea) were purchased from the Republic of Korea. The QuantiChrom^TM^ nitric oxide assay kit (D2NO-100) and peroxide assay kit (DIOX-250) were purchased from BioAssay Systems (Hayward, CA, USA). An AlamarBlue assay kit (DAL1025, Invitrogen, Waltham, MA, USA) was used for cell viability analysis.

### 2.2. Cell Culture

The H460 and A549 human lung adenocarcinoma cell lines were cultured in RPMI-1640 (LM011-51; Welgene, Gyeongsan-si, Republic of Korea), while MRC5 normal lung fibroblasts cells were cultured in DMEM (LM001-05; Welgene, Gyeongsan-si, Republic of Korea) supplemented with 10% FBS and 1% penicillin/streptomycin. All cell lines were purchased from the Korean Cell Line Bank (KCLB, Seoul, Republic of Korea). The cells were sub-cultured every 2–3 days and seeded at different volumes; most of the cells were seeded in 6-well plates at 2 × 10^5^ cells/mL and then incubated for two days at 37 °C with 5% CO_2_.

### 2.3. Plasma Device Setup

**Figure 1:**
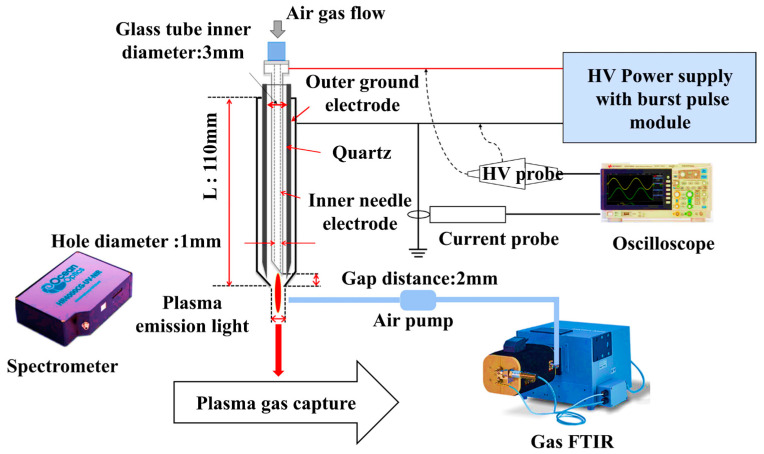
Experimental setup of the nonthermal biocompatible pressure plasma jet (NBP-jet) for treating cell culture media.

NBP-jet plasma (soft jet air plasma) is advantageous for localized sample processing and the generation of reactive species. The plasma temperature and density can be calculated via a nitrogen collisional radiation (CR) model with a spectrometer (HR4000CG-VIS-IR, Ocean Optics Co, Seoul, Republic of Korea) for optical emission spectroscopy (OES). Additionally, gas-FTIR (Fourier transform infrared, MATRIX-MG5 in Bruker Co, Seoul, Republic of Korea) optical absorption spectroscopy (OAS) can be used to measure the concentrations of plasma reactive species such as reactive oxygen species (ROS) and reactive nitrogen species (RNS). The status of the plasma source was monitored via an oscilloscope, spectrometer, and gas-FTIR. Figure 1 illustrates the experimental setup used to monitor the status of the plasma device with an oscilloscope, spectrometer, and gas-FTIR. At the center of the setup is the plasma device, which generates a plasma jet. The design of the plasma device includes a central electrode enclosed in a dielectric tube covered with an outer ground electrode. High voltage is applied to the electrode by passing the gas through the body of the tube and making plasma jets at the nozzle. The plasma source is powered by a high-voltage power supply (V_pp_ = 5 kV) with a burst pulse module. Electrical signals from the plasma source, including voltage and current, are observed using an oscilloscope that displays waveform signals essential for analyzing the electrical characteristics of the plasma. On the left side of the diagram, a spectrometer is used to monitor optical emissions from the plasma. This device captures the spectral characteristics (intensity vs. wavelength), allowing for the identification and analysis of different species present in plasma. The Gas-FTIR (Fourier Transform Infrared Spectroscopy) device was used to analyze the gas composition emitted from the plasma. This setup provides a comprehensive system for analyzing the electrical, optical, and gas-phase characteristics of the plasma source, with a well-designed plasma source that includes a central electrode and dielectric tube to effectively generate and control the plasma jet. The air gas was passed through an air pump, and the gas flow rate was 1 L per minute (lpm).

### 2.4. Physicochemical Properties of the Reactive Species

A commercially available QuantiChrom^TM^ NO detection kit was used to measure the total nitrite NO_2_^−^/NO_3_^−^ content in the media [29]. The total H_2_O_2_ concentration was measured via the oxidation of H_2_O_2_ with Fe_2_^+^/Fe_3_^+^ xylenol orange using a QuantiChrom^TM^ peroxide test kit (DIOX-250; Bioassay Systems, Hayward, CA, USA) [30]. According to the manufacturer’s recommended guidelines, a reactive species analysis was performed. A pH probe (Eutech Instruments, Paisley, UK), an ORP30 Tester, and a CON30 Tester (Clean Instruments, Shanghai, China) were used to measure the pH, oxide-reduction potential (ORP), and conductivity of the treated media. The treatment conditions were as follows: cont. (untreated media used as control), p-CA (para-coumaric acid), NBP (media treated with the NBP-jet plasma around 180 s), and p-CA + NBP (para-coumaric acid combined with 180 s of the NBP-jet plasma-treated media).

### 2.5. Cell Viability

The viability of two lung cancer cell lines (H460 and A549) and one normal lung cell line (MRC5) was examined via the AlamarBlue assay (Invitrogen, Thermo Fisher Scientific, Waltham, MA, USA). The experiments were carried out in triplicate in 96-well plates with the control and treated groups. The cells were seeded at a density of 1 × 10^4^ cells/mL. This study included three different treatment groups to assess cell viability. Initially, the cells were treated with a 0.78–100 µg/mL dilution of p-CA alone. On the other hand, cell culture media was treated with the NBP-jet plasma for approximately 15 to 300 s before being added to the cells. Furthermore, the cells were exposed to both the p-CA and NBP-jet plasma. In the co-treatment, 6.25 and 12.50 µg/mL p-CA were mixed with the NBP-jet plasma-treated media. The treated cells were subsequently incubated at 37 °C with 5% CO_2_. The cells were dyed with AlamarBlue, and the absorbance was measured at 570 nm (Biotek, Winooski, VT, USA) the next day.

### 2.6. Cell Movement Assay

Based on the viability findings, four treatment conditions were employed in the cell movment assay: (1) control (untreated media), (2) para-coumaric acid (6.25 µg/mL p-CA solution), (3) NBP (media treated with a non-thermal biocompatible plasma jet for 180 s), and (4) p-CA + NBP (6.25 µg/mL p-CA combined with media treated with a non-thermal biocompatible plasma jet for 180 s). Cell movement was investigated from the wound healing scratch test. After the wound area was scraped, the cells were washed with PBS, and the treated media was added. Wound closure was observed and recorded under a microscope (Nikon Eclipse Ti, Shinjuku, Japan) at intervals of 0, 12, and 24 h.

### 2.7. Clonogenic Assay

A colony formation assay was performed according to a previous study [31]. In brief, 2 × 10^4^ lung cancer cells were seeded in 35 mm dishes. Following 24 h of treatment, the cells were trypsinized and reseeded at 150 cells/mL in triplicate in 60 mm dishes. After being cultured for up to twelve days, the colonies were measured using ImageJ (version: 1.54j) and manually evaluated in a blinded manner.

### 2.8. Intracellular ROS/RNS Detection

The H460, A549, and MRC5 cells (5 × 10^4^ cells/mL) were seeded in 12-well plates and incubated for 24 h at 37 °C with 5% CO_2_. After 24 h, the cells were subjected to various treatment conditions (cont., p-CA, NBP, and p-CA with NBP). The following day, the cells were washed with PBS and stained with 10 μM DAF-FM DA (D23844, Thermo Fisher Scientific, Waltham, MA, USA) to measure the number of intracellular RNS. Similarly, 100 μM H_2_DCFDA (D399, Thermo Fisher Scientific, Waltham, MA, USA) was used for the detection of ROS. An Olympus confocal microscope (Shinjuku City, Tokyo, Japan) was used to capture images. A stock solution of 10 µM H_2_DCFDA and DAF-FM DA was diluted with DMSO and then mixed with PBS.

### 2.9. Cell Death Analysis by Propidium Iodide (PI) Staining

In a 12-well plate, 5 × 10^4^ cells/mL cells were seeded for cell death analysis. Next, the cells were treated with plasma containing p-CA under several conditions: Cont., p-CA, NBP, and p-CA with NBP. After 24 h of incubation, the cells were washed with PBS, fixed with 4% paraformaldehyde for one hour, and incubated with 100 μL of diluted propidium iodide for 30 min in the dark. Images were taken with an Olympus fluorescence microscope (Shinjuku City, Tokyo, Japan).

### 2.10. Flow Cytometry

In 6-well plates, cells were seeded at a density of 2 × 10^5^ to evaluate the amount of cell death under various treatment conditions. Following treatment, the cells were allowed to incubate for 24 h. A fluorescein isothiocyanate (FITC) Annexin V Apoptosis Detection Kit (BD 556547, Seoul, Republic of Korea) was used to resuspend the pellet in PI and Annexin staining solution, which was then incubated for 15 min on ice. After that, cell death was analyzed via a flow cytometer (BD Bioscience FACSVerse system, Seoul, Republic of Korea). For immunofluorescence staining, the H460 and A549 cells were incubated for 40 min in the dark with a 1:500 ratio of GPX4. After that, the cells were washed with PBS, and Alexa Fluor 488 (A11009, Invitrogen, MA, USA) was added in accordance with the manufacturer’s instructions. Following cell staining, the samples were collected and subjected to instant analysis using a FACSVerse apparatus (BD Biosciences, Seoul, Republic of Korea).

### 2.11. qRT-PCR

RNA was extracted in accordance with the manufacturer’s instructions using the Trizol reagent (Takara Bio Inc., Kusatsu, Japan). RNA concentration was measured using the Synergy HTX multileader (Biotek, Winooski, VT, USA).

cDNA was generated using the Superscript II reverse transcriptase kit (Takara Bio, Inc, Kusatsu, Japan). IQ SYBR Green supermix (BD Biosciences, Seoul, Republic of Korea) was used in real-time PCR utilizing CFX96TM RealTime equipment (BioRad laboratory, Hercules, California, USA). The primers were purchased from Oligo DT (Hanam City, Gyeonggi, Republic of Korea), which was used to create the primers (Table 1). The house-keeping gene Glyceraldehyde 3-phosphate dehydrogenase (GAPDH), which was expressed as a ratio to the control, was used as the control to normalize the results.

### 2.12. Live/Dead Cell Staining

Cell stability was evaluated over a 24 h period after treatment by using a live/dead viability test (Molecular Probes, Invitrogen, Eugene, Oregon, USA) in accordance with the instructions provided by the manufacturer. After the cells were incubated with 0.05% 4 mM Calcein-AM and 0.2% BOBO-3 iodide for 120 min at room temperature, images were taken using a confocal laser scanning microscope.

### 2.13. Cancer Sphere Formation

To make a 2% methylcellulose solution, 6 g of methylcellulose (SIGMA, Steinheim am Albuch, Germany) was stirred with 250 mL of RPMI media at 60 °C for 20 min, added to another 250 mL of media, and incubated at 4 °C overnight. The following day, the solution was centrifuged twice at 4000× *g* for 99 min, and the upper portion was collected and stored at 4 °C. Furthermore, 1.5 g of bacterial-grade agar (GIBCO, Thermo Fisher Scientific GmbH, Dreieich, Germany) was dissolved in 100 mL of H_2_O to make a 1.5% agar solution, which was then autoclaved. Twenty-four-well plates were coated with agar solution and incubated in an incubator for solidification. To generate spheroids, after trypsinization, the pellets were collected, and the cells were resuspended in 20% Methocel solution. A total of 20 μL of this cell suspension was pipetted on a 90 mm dish lid with 10 mL and inverted to form hanging drops. After 24 h, the spheroids were transferred to 24-well plates precoated with agar via a cut 20 μL pipette tip, with one spheroid per well in 1 mL of media. Fluorescence images were taken using an Olympus fluorescence microscope.

### 2.14. Immunostaining

After 24 h of treatment, the cells were washed with PBS and fixed with 4% paraformaldehyde for approximately 30 min. Subsequently, the samples were blocked with 5% BSA in 1× TBST overnight at 4 °C. The following day, the cells were incubated with the primary antibody anti-phospho-p38 (phospho T180 + Y182,) (ab45381, 1:200; Abcam, Cambridge, UK) and GPX4-Alexa Fluor 488 (A11009, 1:500; Invitrogen, Eugene, OR, USA). After washing three times with 1× TBST, secondary antibodies (Alexa Fluor 532-conjugated goat anti-rabbit, A11009, Waltham, Massachusetts, USA, 1:500) and DAPI (1:1000) were added, and the samples were incubated for 2 h at room temperature. Confocal microscopy was used to capture images.

### 2.15. Intracellular Chelate Iron

By using Phen Green SK (Thermo Fisher, P14313, Waltham, Massachusetts, USA), intracellular chelated iron levels were determined in H460 and A549 cells. In brief, after a 24 h treatment period, the cells were incubated with 1 μM Phen Green SK (green) for 15 min at 37 °C. Then, a confocal microscope was used to capture images of the cells.

### 2.16. Determination of Labile Iron Pool (LIP)

Labile iron pool (LIP) levels were measured using spectrofluorometry following the method provided in [32] with slight modifications. Briefly, 1 × 10^4^ cells were seeded and given treatment with the p-CA and NBP-jet plasma. After 24 h, the cells were suspended in 2 mL of pre-warmed buffer containing 20 mM of 2-hydroxyethyl-1-piperazinepropanesulfonic acid (HEPES) (pH 7.3), 150 mM NaCl, and 1% bovine serum albumin (BSA). The cells were then incubated with 50 mM Calcein-AM for 20 min at 37 °C. After incubation, cells were centrifuged, washed, and resuspended in a fresh buffer. The LIP levels were quantified using excitation and emission wavelengths set at 483 nm and 505 nm, respectively.

### 2.17. Lipid Peroxidation Assay

Following the manufacturer’s (Cayman, Chemical, Ann Arbor, MI, USA) instructions, lipid peroxidation induced by co-treatment was quantified via the Thiobarbituric Acid Reactive Substances (TBARS) assay. In a glass test tube, 100 μL of sodium dodecyl sulfate (SDS) solution was mixed with 100 μL of homogenized cancer cells from each group after treatment. The solutions were heated to boiling for 50 min with 4 mL of color reagent. After cooling, the solution was centrifuged at 3500× *g* for 15 min. The organic phase of the supernatant was then analyzed with a spectrophotometer at 530 nm for excitation and 550 nm for emission. Malondialdehyde (MDA) levels were measured using the following formula to detect lipid peroxidation levels:

$$MDA\;(µM)=\frac{\left(Corected\;fluorescence\right)-(y\;intecept)}{slope}$$

### 2.18. GSH Assay

Following a 24 h treatment period, the cells were collected and homogenized with 10 µM Tris-HCl buffer (pH 7.4). Then, the samples were centrifuged at 15,000× *g* for 10 min. After that, the supernatant was collected, and as instructed, a glutathione (GSH) assay (A006-2-1, Nanjing Jiancheng Bioengineering Institute, Nanjing, China) was performed. After a 30 min incubation period, the samples were read at 405 nm with a spectrophotometer.

### 2.19. Western Blot Analysis

Following treatment, the cancer cells were harvested, and the entire protein was separated using Radioimmunoprecipitation assay (RIPA) lysis buffer (Tech-Innovation, Seoul, Republic of Korea) for ten minutes on ice. The cell lysate was centrifuged at 13,000× *g* for 30 min at 4 °C, and the protein concentration in each supernatant was then determined using a BSA protein assay kit (Thermo Fisher Scientific, Waltham, Massachusetts, USA). Blotting involved decentralizing the total protein using 4–15% sodium dodecyl sulfate-polyacrylamide gel electrophoresis (SDS–PAGE) (Bio-Rad, Hercules, California, USA) and transferring it onto nitrocellulose membranes. After blocking the membrane bolts with 5% BSA for two hours, the membrane bolts were probed overnight with specified primary antibodies, including GPX4, NRF2, SLC7A11, JNK, P-JNK, P-ERK, ERK, p53, p-p38, p38, and GAPDH (Cell Signaling Technology, Danvers, Massachusetts, USA). For an additional hour, the membrane was incubated with the corresponding anti-rabbit/mouse IgG secondary antibody that was coupled with horseradish peroxidase (Cell Signaling Technology, USA). The protein band was seen using Clarity MaxTM Western ECL substrate (Bio-Rad, Milan, Lombardy, Italy), and the target protein expression levels were normalized by the house-keeping protein (GAPDH), which was used as a loading control.

### 2.20. Statistical Analysis

The means and standard deviations represent the results of three individual trials. Student’s *t*-test was utilized to determine the statistical significance of the data points, and the significant differences are denoted by * *p* ≤ 0.05, ** *p* ≤ 0.01, and *** *p* ≤ 0.001 versus the untreated control.

## 3. Results

### 3.1. Measurement of the Electrical Discharge Power

**Figure 2:**
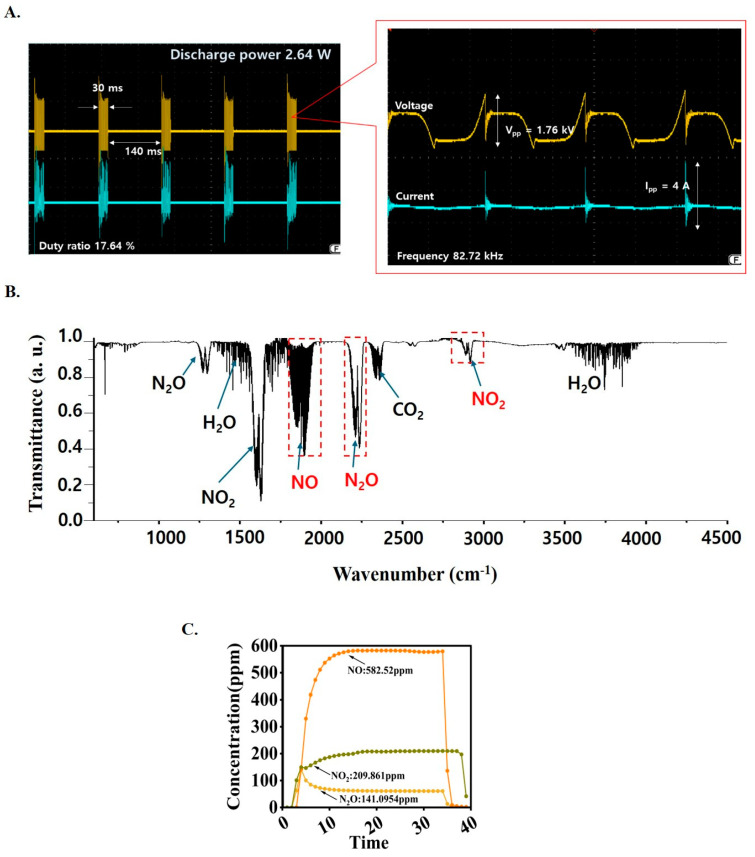
Plasma diagnostics of the NBP-jet: (**A**) Discharge voltage and current of the NBP-jet plasma. (**B**) Total absorption spectra of NBP-jet plasma measured by using gas-FTIR. (**C**) Time-dependent gas concentrations of the NBP-jet plasma.

The electrical signal, discharge voltage, and current were measured using an oscilloscope equipped with high-voltage (1:1000) and current probes as shown in Figure 2A. The discharge power was calculated using a formula that includes the integral of the time-dependent measured voltage and current for the applied frequency, as revealed in Equation (1).

(1) $$P=freqency\times \int_{0}^{T}V(t)\cdot I\left(t\right)dt$$

The air plasma jet (NBP-jet) had an input voltage with a burst pulse ON duty ratio of 17.64% (Pulse OFF time: 30 ms; Pulse ON time: 140 ms), as shown in Figure 2A, which also shows an enlarged view of the signal during the pulse-on period. The measured frequency was 82.72 kHz, as shown in Figure 2A. This burst pulse includes a resting period, which helps cool the electrode by reducing heat. This approach was selected due to the electrode’s vulnerability to high temperatures. In this case, the peak-to-peak discharge voltage and current were measured at 1.76 kV and 4.0 A, respectively, as shown in Figure 2A. The discharge power was calculated to be 2.64 W according to Equation (1). In Figure 2A, a sharp spike-shaped curve is observed, indicating a voltage drop caused by nonthermal plasma discharge, accompanied by a sudden surge in current.

### 3.2. Measurement of the Plasma Temperature and Density

Physical properties, such as plasma temperature and density, are crucial for assessing nonthermal plasma status. Most atmospheric pressure plasmas contain excited nitrogen molecules due to electron impact excitation in ambient air. This phenomenon was demonstrated by the optical emission spectra of the excited nitrogen species. The emission spectrum of the nitrogen plasma was measured using an optical spectrometer in the generated air plasma.

A detailed explanation of the plasma temperature and density is provided in Supplementary Materials Section S1 [33,34,35,36,37,38,39] and Figure S1A–C.

### 3.3. Measurement of the Plasma Reactive Species Concentration

Fourier-transform infrared (FTIR) spectroscopy is a powerful analytical technique widely used for the qualitative and quantitative analysis of reactive gases in plasma. This method leverages the interaction of infrared radiation with molecular vibrations to identify and quantify different chemical species in a gas sample.

Our gas-FTIR (model Matrix-MG5 of Bruker co, Seoul, Republic of Korea) has a spectral range between 4521 and 582 cm^−1^ (2.21~17.18 μm) for a spectral resolution of 1 cm^−1^. This equipment can measure time-dependent concentrations of N_2_O, NO, NO_2_, O_3_, and HNO_3_. The results of the gas-FTIR measurements are shown in Figure 2B. Figure 2B presents the total absorption spectra of the reactive species generated by air plasma. CO_2_ and H_2_O are typical absorption spectrum signals measured in ambient air. After plasma discharge, NO, N_2_O, and NO_2_ were detected. These molecular spectral bands of Figure 2B in air plasma can be verified with reference to [40] and https://vpl.astro.washington.edu/spectra/ (accessed on 28 March 2025). The concentration values were calculated using the NO, NO_2_, and N_2_O data within the region marked by the red box in Figure 2B. This study also monitored changes in the gas concentration over time, as illustrated in Figure 2C. This figure depicts the temporal evolution of the concentrations of NO, N_2_O, and NO_2_ during the plasma discharge process of NBP-jet plasma. The NO concentration exhibited sharply increased and then plateaued after approximately 10 min. The N_2_O concentration initially increased but then decreased and stabilized, forming a steady curve. The NO_2_ concentration gradually increased but remained below 40% of the NO concentration throughout the measurement period. The NO concentration reached approximately 583 ppm in the NBP-jet. The NO_2_ and N_2_O concentrations were approximately 209 and 141 ppm, respectively, as revealed in Figure 2C. Therefore, it is expected that the concentration of reactive species is responsible for killing the cancer cells.

### 3.4. Physiochemical Properties of NBP-Jet Plasma-Treated Media

**Figure 3:**
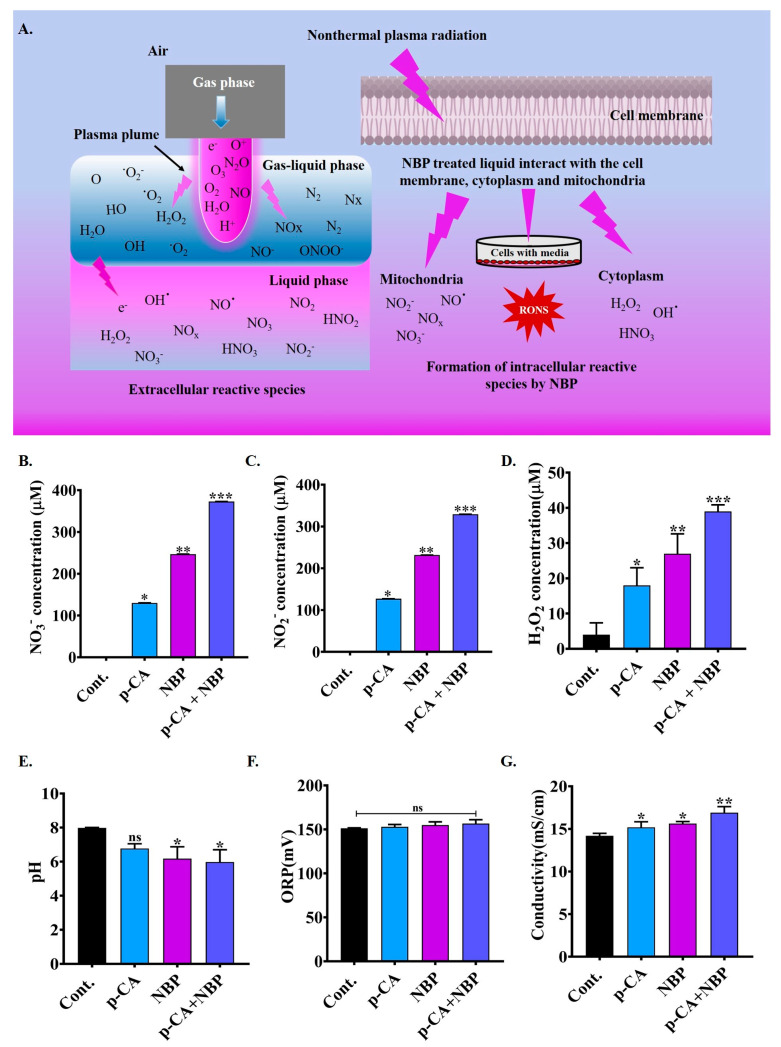
Physicochemical characteristics of cell culture media after para-coumaric acid with NBP-jet plasma treatment: (**A**) Extracellular and intracellular reactive species are produced by the nonthermal biocompatible plasma (NBP) jet. (**B**) NO_3_^−^ concentration. (**C**) NO_2_^−^ concentration. (**D**) H_2_O_2_ concentration. (**E**) pH. (**F**) Oxidation-reduction potential (ORP). (**G**) Conductivity after combination treatment. The data are presented as the means ± standard deviations of three independent experiments. Significance levels are denoted as * *p* < 0.05, ** *p* < 0.01, and *** *p* < 0. 001 vs. the control/treated group. ns, not significant.

The NBP-jet plasma interacts with various gases in the air, including oxygen, nitrogen, and water, which leads to the formation of short-lived reactive species such as nitric oxide, ozone, hydroxyl radicals, singlet oxygen, and superoxide anions, as shown in Figure 3A. These species form extracellular reactive species by converting the nitrogen and oxygen at the air–liquid interface to the reactive atoms dissolving into the liquid. As shown in Figure 3A, long-lived species in the plasma-treated media, such as hydrogen peroxide, nitrite, and nitrate, interact with the mitochondria, cytoplasm, and cell membrane to increase the levels of intracellular reactive oxygen and nitrogen species (RONS). The physicochemical characteristics of the treated media are shown in Figure 3B–G. The combination of para-coumaric acid and NBP-jet plasma (p-CA + NBP) produced higher levels of NO_3_^−^ (373 μM) than the NBP plasma (247 μM) and para-coumaric acid (130 μM) alone, as shown in Figure 3B. In addition, Figure 3C shows a higher concentration of nitrite (NO_2_^−^) (329.27 μM) in the co-treatment group than in the para-coumaric acid (127.15 μM) and NBP plasma (231.70 μM) alone. The H_2_O_2_ concentration was approximately 39 μM in the para-coumaric acid with the NBP-jet plasma-treated media, whereas it was 4 μM in the control, 18 μM in the para-coumaric acid, and 27 μM in the NBP plasma, as shown in Figure 3D. In contrast, as shown in Figure 3E, the pH values decreased more in the co-treatment group (5.9) than in the para-coumaric acid (6.7) and NBP plasma (6.17) alone. ORP slightly increased in the para-coumaric acid alone, NBP-jet plasma alone, and para-coumaric acid with NBP-jet plasma-treated groups to 152.9, 154.9, and 156.6 mV, respectively, from the control (151.2 mV), as shown in Figure 3F. Furthermore, compared with those of the control (14.2 mScm^−1^), the conductivity values for the para-coumaric acid, NBP-jet plasma, and para-coumaric acid with NBP-jet plasma were increased to 15.1, 15.6, and 16.9 mScm^−1^, respectively, as shown in Figure 3G.

### 3.5. NBP-Jet Plasma and Para-Coumaric Acid Treated Media Decreased Cell Viability, Colony Formation and Movement Ability of Lung Adenocarcinoma

**Figure 4:**
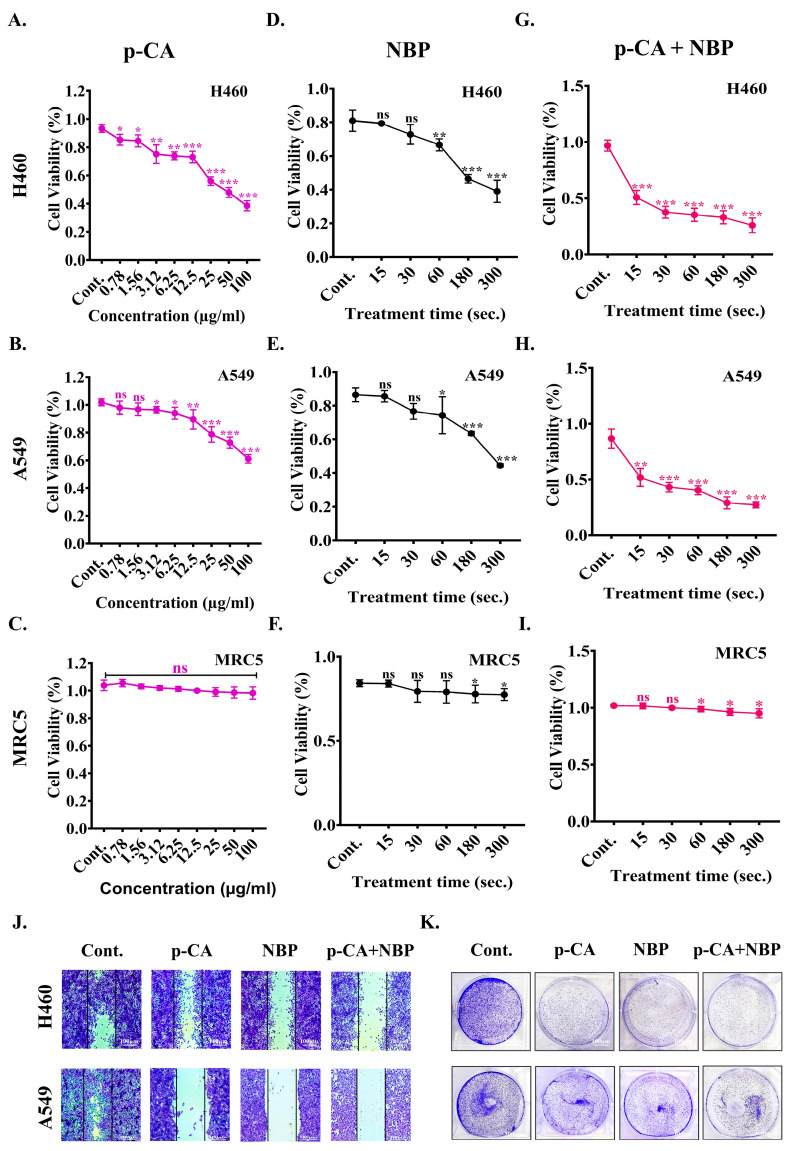
Effect of combined treatment (para-coumaric acid and NBP-jet plasma) on cell viability, colony formation and migration in H460, A549, and MRC5 cells: Cell viability of para-coumaric acid alone at various doses (0–100 µg/mL) for 24 h on (**A**) H460 cell, (**B**) A549 cell, and (**C**) MRC5 cell. Effect of NBP-jet plasma alone exposure for durations of 15, 30, 60, 180, and 300 s on (**D**) H460 cell, (**E**) A549 cell, and (**F**) MRC5 cell. Combination of para-coumaric acid (6.25 µg/mL) and NBP-jet plasma (15–300 s exposure) on (**G**) H460 cell, (**H**) A549 cell, and (**I**) MRC5 cell viability. (**J**) Cell movement assay of H460 and A549 cells at 6.25 µg/mL of para-coumaric acid with 180 s of NBP-jet plasma treatment after 24 h incubation. (**K**) Colony formation assay at 6.25 µg/mL of para-coumaric acid with 180 s of NBP-jet plasma treatment after 24 h incubation. The scale bar represents 100 µm. The data are presented as the means ± standard deviations of three independent experiments. Significance levels are denoted as * *p* < 0.05, ** *p* < 0.01, and *** *p* < 0.001. ns, not significant.

To investigate the potential anticancer effects of para-coumaric acid (p-CA), human lung adenocarcinoma cells (H460 and A549) and normal lung cells (MRC5) were treated with para-coumaric acid alone at various concentrations (0–100 μg/mL) for 2 and 24 h, as shown in Figure S2A–C and Figure 4A–C. The 24 h incubation period was chosen for further experiments. Para-coumaric acid alone exhibited minimal toxicity, approximately 40%, 60%, and 92%, at high concentrations (100 µg/mL) in the H460, A549, and MRC5 cells, respectively (Figure 4A–C). In addition, this study also evaluated cell viability by using the NBP-jet plasma alone. The NBP-jet plasma-treated media reduce cell viability by approximately 48% (H460) and 51% (A549) and have mild toxicity in normal cells of 91% (MRC5) at 300 s, as shown in Figure 4D–F. Then, we exposed the cells to the NBP-jet plasma combined with the para-coumaric acid at doses of 6.25 and 12.5 µg/mL. The NBP-jet treatment duration was 15 to 300 s. Notably, when we applied co-treatment, the cell viability decreased in all the treated groups, reflecting an inhibitory concentration of 20% lung adenocarcinoma cell death, as shown in Figure 4G–H and Figure S2D–F. However, the H460 cells were more sensitive than the A549 cells were to this co-treatment, which also led to changes in cell morphology (Figure S3A,B). Furthermore, 6.25 µg/mL of para-coumaric acid and 180 s of the NBP-jet plasma treatment were utilized for entire experiments. In this study, cell movement was also observed via crystal violet assays. Compared with the control, the metastatic potential of lung adenocarcinoma cells was reduced in all the treated groups, as shown in Figure 4J and Figure S3C. Co-treatment significantly inhibited the ability of the H460 and A549 cells to form colonies, as shown in Figure 4K.

### 3.6. NBP-Jet Plasma and Para-Coumaric Acid Induce Apoptosis by Inhibiting Cancer Spheroid Formation Through the MAPK and PARP Pathways

The NBP-jet plasma and para-coumaric acid significantly increased the intracellular RONS levels in the H460 and A549 cells, as indicated by the fluorescent probes H_2_DCFDA and DAF-FM (Figure S4). As shown in Supplementary Figure S4A,B, the amounts of ROS were 2.94% (control), 14.79% (para-coumaric acid), 27.53% (NBP), and 60.25% (para-coumaric acid with NBP) in the H460 cells. In contrast, in the A549 cells, the percentages were 4.29% (control), 10.73% (para-coumaric acid), 28.51% (NBP), and 45% (para-coumaric acid with NBP). However, co-treatment exhibits higher intracellular ROS levels compared to the para-coumaric acid and NBP plasma alone. In addition, co-treatment showed more RNS levels in both cell lines, including 13.92% (H460) and 10.72% (A549), as shown in Figure S4A,B. Compared to the single treatments, co-treatment did not adversely decrease RONS levels, indicating increased oxidative stress in both adenocarcinoma cells (Figure S4A,B).

Furthermore, to evaluate the proapoptotic effects of the para-coumaric acid and NBP-jet plasma, we performed a cell death assay via flow cytometry. The results showed that co-treatment was also able to induce apoptosis, with higher rates of early and late apoptosis, as shown in Figure S5A,B. In the H460 cells, the percentage of apoptotic cells was 0.08% in the control group, 45.04% in the para-coumaric acid group, 53.35% in the NBP plasma group, and 85.32% in the para-coumaric acid and NBP plasma group (Figure S5). Minimal necrosis, including 0.07% (control), 1.17% (para-coumaric acid), 4.49% (NBP), and 5.52% (co-treatment), was observed in the H460 cells. Similarly, in the A549 cells, 0.00% apoptosis was detected in the control group, whereas 38.28%, 53.1%, and 76.12% in the para-coumaric acid, NBP, and co-treatment, respectively (Figure S5). The A549 cells were less susceptible to co-treatment than the H460 cells. These findings suggest that adenocarcinoma cells produce more RONS after exposure to co-treatment.

**Figure 5:**
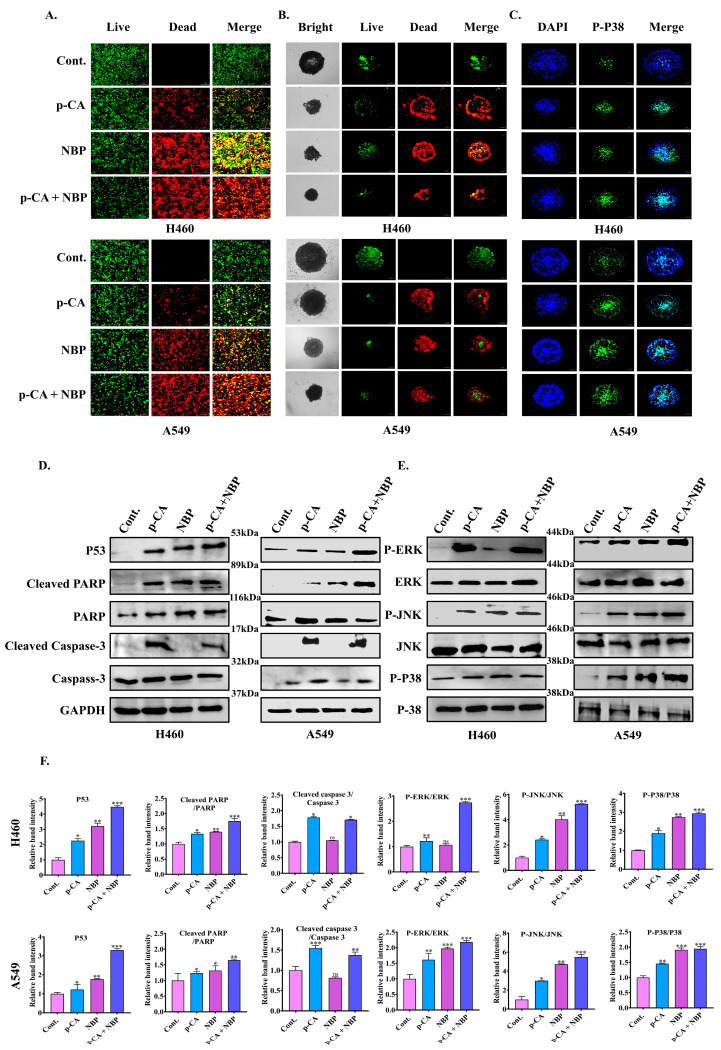
Induction of apoptosis-related cell death in lung adenocarcinoma by the para-coumaric acid with NBP-jet plasma: (**A**) Confocal microscopy image of the H460 and A549 cells by using live/dead assay. (**B**) Spheroid growth was reduced by combination treatment in the H460 and A549 cell lines. The green fluorescence indicates live cells, while the red fluorescence represents dead cells, providing a visual assessment of cell viability. (**C**) Analysis of membrane damage by immunofluorescence staining in the H460 and A549 cells. The cell nucleus was observed with DAPI (blue) staining, and cell membrane damage was identified using p-p38 staining (green). (**D**) Apoptosis-related protein analysis by Western blot in the H460 and A549 cells after 24 h of treatment with para-coumaric acid (6.25 µg/mL) and NBP-jet plasma. (**E**) MAPK signaling pathway-related proteins, including phosphorylated ERK, JNK, and p-p38, following co-treatment in the H460 and A549 cells. (**F**) Quantification of apoptosis and MAPK-related proteins (p53, Cleaved PARP, Cleaved Caspase 3, P-ERK, P-JNK, and p-p38) in the H460 and A549 cells using the ImageJ software. The house-keeping protein GAPDH (~37 kDa) was used as a loading control. The data are presented as the means ± standard deviations of three independent experiments. Significance levels are denoted as * *p* < 0.05, ** *p* < 0.01, and *** *p* < 0. 001 vs. the control/treated group. ns, not significant. The scale bar represents 100 µm. Original western blots can be found at Supplementary Materials.

Additionally, live/dead co-stained H460 and A549 cells exhibit more cell death in co-treatment than in treatment alone (Figure 5A). As shown in Figure 5B, the co-treatment decreased the size of the tumor spheres more than the para-coumaric acid and NBP plasma alone in both cell lines. Supplementary Figure S6A–C illustrate a time-dependent reduction in spheroid size following co-treatment. Furthermore, spheroid viability significantly decreased in both cancer cell types, as shown in Figure 5B. Confocal images taken at a 10 µm focal depth revealed live cells predominantly in the center of the spheroids rather than on the surface (live/dead image), suggesting that the adenocarcinoma cells were going die in both cell lines.

Immunofluorescence staining was performed to determine the exact pathways involved. Co-treatment induced cell membrane damage by increasing the intensity level of p-p38 (Figure 5C and Figure S6D,E) in the H460 and A549 cells. The MAPK pathway, particularly p-p38, plays a critical role in oxidative stress, and p53 acts as a sensor of cellular energy involved in regulating the energy stress response (Figure 5C–F). Quantitative real-time polymerase chain reaction (qRT-PCR) was employed to investigate how combination treatment affects the expression of the genes associated with apoptosis in the H460 and A549 lung cancer cells (Figure S7A–H). p53, which regulates cell cycle arrest and cell death induction, was significantly increased in both cancer cell lines after co-treatment (Figure S7A,E). The upregulation of PARP, Caspase-3, and Bax was also observed in both H460 and A549 cells (Figure S7B–D,F–H).

Surprisingly, Western blotting (Figure 5D–F) confirmed that increased levels of apoptosis regulatory proteins, such as p53, p-p38, PJNK, PERK, Caspase-3, and PARP, were activated more by single treatment than by co-treatment. However, apoptosis does not occur via the Caspase-3 and JNK pathways, which play major roles in apoptotic cell death through p53. Therefore, we are moving toward understanding ferroptotic cell death.

### 3.7. Combination Treatment Induced Ferroptotic Cell Death via the Downregulation of Ferroptosis Markers in Lung Adenocarcinoma Cell Line

**Figure 6:**
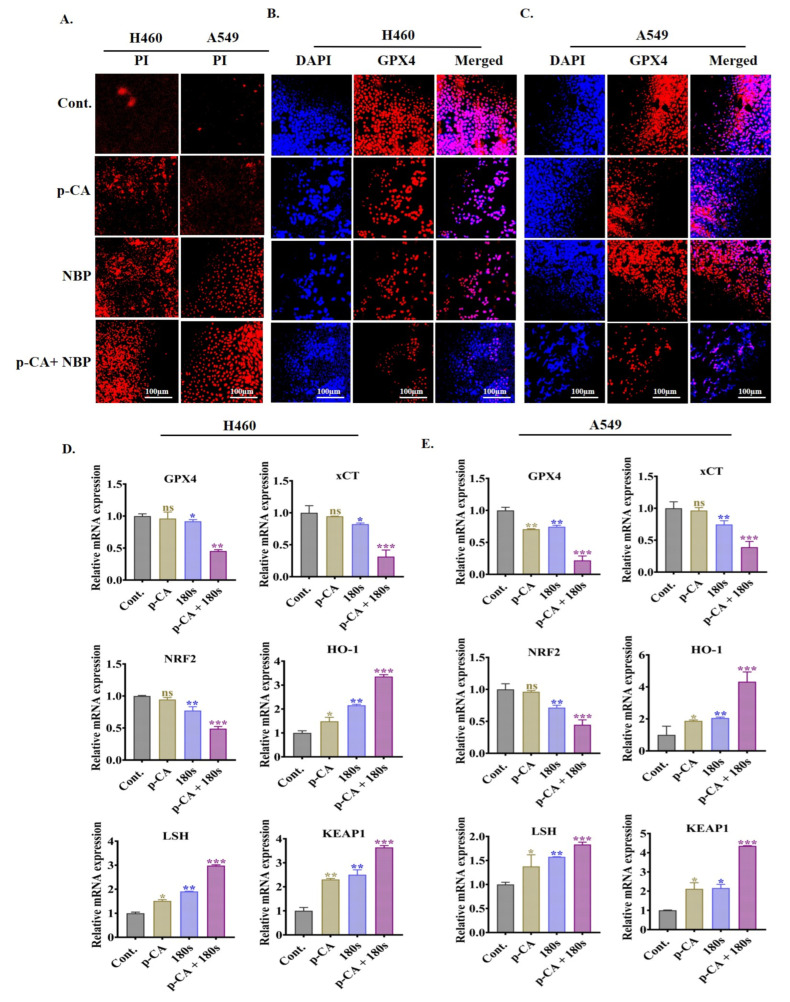
NBP-jet plasma combined with para-coumaric acid triggers ferroptosis in lung adenocarcinoma cell lines. The cells were treated with the NBP-jet plasma (180 s) and para-coumaric acid (6.25 µg/mL): (**A**) Assessment of cell membrane damage using PI staining. (**B**,**C**) Immunofluorescence staining of GPX4 (red) and DAPI (blue) were used as markers for the nucleus after 24 h of treatment in H460 and A549 cells. (**D**) mRNA expression levels of ferroptosis-related genes, namely, GPX4, xCT, NRF2, HO-1, LSH, and KEAP-1, in the H460 cells were measured via qRT-PCR. (**E**) mRNA expression levels of ferroptosis related genes, namely, GPX4, xCT, NRF2, HO-1, LSH, and KEAP-1, in the A549 cells were measured via qRT-PCR. The scale bar in the images represents 100 µm. The data are presented as the means ± standard deviations of three independent experiments. Significance levels are denoted as * *p* < 0.05, ** *p* < 0.01, and *** *p* < 0. 001 vs. the control/treated group. ns, not significant.

As shown in Figure 6A, membrane damage was determined by PI staining in both the H460 and A549 cells, including the control, para-coumaric acid alone, NBP plasma alone, and the combination of para-coumaric acid with NBP plasma. Compared with the single treatments, the combination treatment resulted in greater PI intensity, indicating enhanced membrane damage (Figure 6A). To determine whether combination treatment induces ferroptosis, we examined the intracellular chelatable iron and LIP level (Figures S8 and S9) using Phen Green SK fluorescence and Calcein-AM spectrophotometry, respectively. Reactive elements, such as iron, have the potential for numerous biological functions, including ferroptosis. Co-treatment significantly reduced the percentage of Phen Green SK fluorescence intensity, indicating increased iron production inside the cells, as shown in Figure S8A–D [41]. Notably, the LIP levels were elevated, as confirmed by spectrophotometry analysis (Figure S9A,B). Immunofluorescence staining was used to confirm the presence of DNA double-strand breaks, and the results revealed that both cell lines presented reduced GPX4 intensity. DAPI and GPX4 staining were performed to evaluate ferroptotic cell death due to membrane damage induced by the NBP plasma with para-coumaric acid, as shown in Figure 6B,C. Compared with the control, the para-coumaric acid and NBP plasma alone slightly decreased the GPX4 intensity in both cell lines, as shown in Figure 6B,C. Interestingly, co-treatment significantly reduced GPX4 intensity in both lung adenocarcinoma cell lines. By decreasing the GPX4 levels, co-treatment increases the DNA damage response through the accumulation of lipid peroxidation products. The qRT-PCR results suggested that co-treatment triggered ferroptosis by downregulating GPX4, xCT, and NRF2 gene expression, while HO-1, LSH, and KEAP-1 expression was upregulated in both cell lines (Figure 6D,E). Multiple studies have confirmed that the induction of ferroptosis involves the upregulation of HO-1 [42] and downregulation of GPX4/xCT/NRF-2 expression [43]. In H460 cells, GPX4 expression decreased by 0.45-fold in the co-treatment group, compared to 0.96-fold with para-coumaric acid alone and 0.90-fold with NBP plasma alone, relative to the control group (Figure 6D). Similarly, Figure 6E shows that co-treatment decreased GPX4 expression (0.2-fold) in the A549 cells more compared to control. These findings indicate that NBP plasma combined with para-coumaric acid not only promotes apoptosis but also triggers ferroptosis by inhibiting cell growth.

### 3.8. NBP-Jet Plasma and Para-Coumaric Acid Trigger Ferroptosis by Modulating the GPX4, xCT and NRF2 Pathways

**Figure 7:**
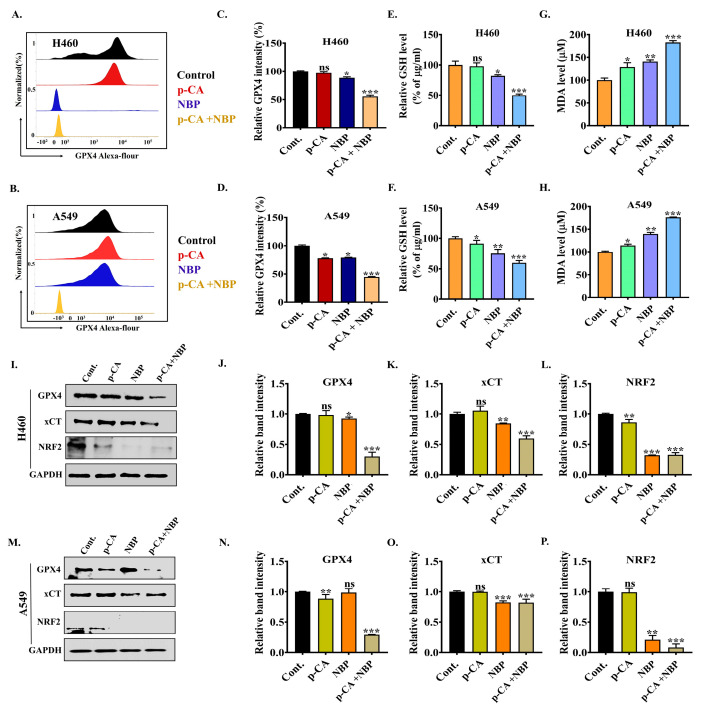
Effects of combined treatment on lung adenocarcinoma cell lines to induce ferroptosis through the GPX4, xCT, and NRF2 signaling pathways: (**A**,**B**) The H460 and A549 cells were treated with the para-coumaric acid and NBP-jet plasma for identifying GPX4 protein level by flow cytometry. (**C**,**D**) Quantification of GPX4 fluorescence intensity in both cell lines using the ImageJ software. (**E**,**F**) GSH levels in the H460 and A549 cells after 24 h of treatment. (**G**,**H**) MDA levels in the H460 and A549 cells following treatment with the para-coumaric acid and NBP-jet plasma for 24 h. (**I**) Ferroptosis-related protein analysis of the H460 cells via Western blotting. Quantification of the band intensities of (**J**) GPX4, (**K**) xCT, and (**L**) NRF2 in the H460 cells. (**M**) Ferroptosis-related protein analysis of the A549 cells via Western blotting. Quantification of the band intensities of (**N**) GPX4, (**O**) xCT, and (**P**) NRF2 in the A549 cells. The data are presented as the means ± standard deviations of three independent experiments. Significance levels are denoted as * *p* < 0.05, ** *p* < 0.01, and *** *p* < 0.001 vs. the control/treated group. ns, not significant. Original western blots can be found at Supplementary Materials.

GPX4 plays a vital role in protecting cells from lipid peroxidation-induced damage by reducing toxic lipid hydroperoxides into nontoxic lipid alcohols [44]. The flow cytometry analysis revealed that GPX4 expression was decreased by the combination treatment, as shown in Figure 7A–D, which indicates a weakened ability of cancer cells to detoxify lipid peroxides. Figure 7C,D also show that co-treatment (0.55-fold and 0.44-fold) has less fluorescence intensity than the para-coumaric acid (0.97-fold and 0.77-fold) and NBP plasma (0.88- and 0.79-fold) alone in the H460 and A549 cell lines. In addition, co-treatment decreased the intracellular GSH level (Figure 7E,F), whereas the MDA level increased (Figure 7G,H), indicating that oxidative stress occurred inside both the lung adenocarcinoma cell lines. The increase in the MDA levels enhances oxidative stress and membrane damage, while the depletion of GSH indicates a reduction in antioxidant capacity, further sensitizing cells to ferroptosis [44,45]. The protein levels of GPX4, xCT, and NRF2 were significantly suppressed by co-treatment (Figure 7I–P) in both the adenocarcinoma cell lines. xCT acts as a crucial component of the cellular antioxidant system by importing cystine, which is subsequently converted to cysteine for GSH synthesis. The downregulation of xCT expression reduces cystine uptake, leading to inhibited GSH biosynthesis and consequently decreased GPX4 activity. Figure 7K,O also show that the co-treatment significantly suppressed the xCT band intensity in both the adenocarcinoma cell lines, indicating a disruption in cystine uptake and the subsequent antioxidant defense. The NRF2 protein levels were also remarkably downregulated, as shown in Figure 7I,L,M,P, by co-treatment, suggesting that an impaired cellular adaptive response to oxidative stress controls xCT transcription to sustain redox balance. A decrease in the expression of NRF2, xCT, and GPX4 synergistically enhances lipid peroxidation and ferroptosis in lung adenocarcinoma cells.

## 4. Discussion

Para-coumaric acid, a phenolic compound, has potential as an anticancer agent, but its poor aqueous solubility and unfavorable pharmacokinetics hinder its clinical value [25]. To increase therapeutic efficacy, several tactics, such as combination treatment, have been suggested. Para-coumaric acid and nanoparticles trigger ferroptosis by inhibiting the GPX4 signaling pathway in chondrocytes [46]. Moreover, naringin combined with para-coumaric acid synergistically induced anticancer efficacy in A431 cells [47].

In this study, we also found that NBP-jet plasma combined with para-coumaric acid increased the effectiveness of para-coumaric acid by promoting lung adenocarcinoma cell death. According to other studies, NBP has significant potential for use in combination with clinical drugs (RSL3) to increase the accessibility of therapeutic targets and improve the overall treatment efficacy [48]. NBP-jet plasma-treated media contain reactive species such as NO, NO_2_^−^, NO_3_^−^, and H_2_O_2_ which influence the pH, ORP, and conductivity, contributing to the anticancer properties shown in Figure 3A–G. A relatively high concentration of charged particles can alter the pH of cell culture media through the following Equation (2):

(2) $${2e}^{-}+{2H}_{2}O\to {2OH}^{-}+H_{2}$$

The analysis of pH in media is crucial in cancer treatment, as it helps to optimize the generation of reactive oxygen and nitrogen species (RONS). The pH value decreased when the conductivity increased (Figure 3E,G) with the co-treatment. However, the effect of co-treatment on the ORP is not well understood (Figure 3F). Under acidic conditions, NBP-jet plasma-treated media can form various nitrite species. These species are produced through the interaction of the plasma plume tip with ambient air. Reactions between singlet oxygen, energetic electrons, and molecular nitrogen result in the formation of reactive oxygen and nitrogen species (RONS), such as HNO_2_, HNO_3_, and ONOOH, as revealed in Reactions (3)–(9) [49,50].

(3) $$e^{-}+N_{2}\to N_{2}^{*}+e^{-}$$

(4) $$N_{2}^{*}+O\to NO+N^{*}$$

(5) $$NO+O\to N_{2}O$$

(6) $$2NO_{2}+H_{2}O\to HNO_{2}+HNO_{3}$$

(7) $$NO+•\;OH\to {HN}_{2}O$$

(8) $${NO}_{2}+•\;OH\to {HNO}_{3}$$

(9) $$HNO_{2}+H_{2}O_{2}\to ONOOH+H_{2}O$$

NO, a reactive diatomic free radical, can increase cellular RONS levels, leading to the nitrosylation and oxidation of cellular components [51]. These reactive species combine with para-coumaric acids to decrease lung adenocarcinoma (H460 and A549) cell viability without causing adverse effects on normal lung cells, as shown in Figure 4G–I. The co-treatment enhances the efficacy of para-coumaric acid by stimulating intracellular RONS levels (Figure S4A,B), whereas cancer cell movement and colony formation are inhibited (Figure 4J,K and Figure S3A–C). These findings suggest that co-treatment induced cell death through the activation of the p53 and PARP pathways, as shown in Figure S7 and Figure 5D–F. Notably, these results are similar to those of previous studies, where co-treatment was shown to stimulate a number of cell death pathways, such as apoptosis [52], autophagy [53], antiaging [54], and pyroptosis [55].

Moreover, ferroptosis is a form of cell death regulated by the xCT-GSH-GPX4 pathway and is characterized by iron and lipid peroxidation. xCT is responsible for the regulation of redox and ferroptosis pathways and drug resistance in multiple cancers via transcription factors, including NRF2 and p53 [56]. In addition, the ferroptosis regulator GPX4 helps detoxify lipid peroxides, while xCT supplies cystine for glutathione synthesis to regulate the NRF2 pathway as a master antioxidant transcription factor. In contrast, apoptosis, identified by caspase activation and DNA fragmentation, is regulated by p53 to promote apoptosis by activating proapoptotic genes and repressing antiapoptotic genes [8]. p53 also modulates ferroptosis through the downregulation of xCT with GSH and GPX4. This finding supports our present study: in Figure 5D–F, the p53 protein expression increased, whereas GPX4, xCT, and NRF2 expression decreased (Figure 7I–P). Moreover, ROS promote apoptosis by activating p-p38 MAPK through mitochondrial dysfunction (Figure 5B–F) and ferroptosis by increasing iron and lipid peroxidation (MDA) levels (Figure S8 and Figure 7G,H). Both of these pathways have been linked to excessive DNA damage during ferroptosis or apoptosis, as it activates PARP. However, the relationship between apoptosis and ferroptosis is still unknown.

Furthermore, mitochondrial damage occurred (Figure 6B,C) by reducing GPX4 levels, whereas PI intensity was increased (Figure 6A) by the combination treatment. A recent study reported that GPX4 contains a crucial selenocysteine residue required to inhibit ferroptosis [57]. Glutathione (GSH) acts as a cofactor for GPX4, which eliminates phospholipid peroxides and prevents the accumulation of lipid-related ROS dependent on iron (Fe^2+^), thereby inhibiting ferroptosis [58]. Several types of tumor cells may undergo ferroptosis and generate toxic lipid ROS if GPX4 activity is inhibited [59], while xCT is an antiporter protein on the cell membrane that controls the homeostasis of glutathione and cysteine in cells. At the transcriptional level, xCT expression is regulated primarily by the p53 and NRF2 proteins [8]. This finding also supports our present study: Figure 6D,E reveal that GPX4, xCT, and NRF2 expression was downregulated whereas HO-1, LSH, and KEAP1 expression was upregulated by co-treatment in both adenocarcinoma cell lines. The downregulation of protein expressions in H460 and A549 cells causes ferroptosis, as shown in Figure 7I–P. Despite this, a variety of studies have shown that xCT is degraded in a proteasome-dependent manner [7].

**Figure 8:**
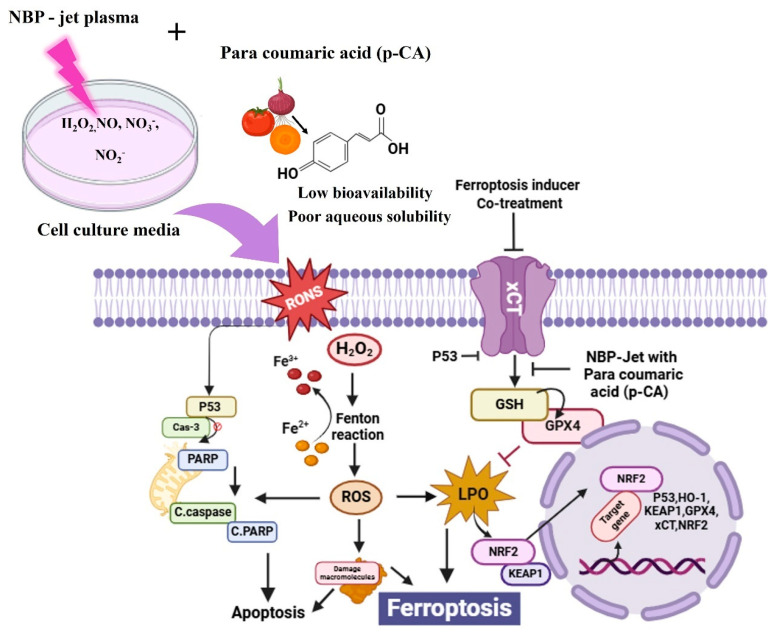
Potential mechanism of lung adenocarcinoma cell death (H460 and A549) after exposure to co-treatment. This treatment increases the intracellular levels of RONS, which results in lipid peroxidation and leads to an alteration in cellular redox homeostasis with consequent induction of cell death.

These investigations are supported present study: NBP-jet plasma with para-coumaric acid enhanced adenocarcinoma cell death. Figure 8 shows that the co-treatment works synergistically to elicit oxidative stress and lipid peroxidation and suppresses GPX4 expression, leading to ferroptosis. In addition, excessive intracellular RONS stimulate lipid peroxidation, which leads to cell stress and alters energy metabolism to induce ferroptosis via the GPX4, xCT, and NRF2 pathways. However, cell death is enhanced by DNA damage through the interaction between ferroptotic and apoptotic processes. Our findings reveal that NBP-jet plasma may increase the availability of para-coumaric acid and ferroptosis sensitivity in H460 and A549 cells, offering novel approaches for the treatment of lung adenocarcinoma.

## 5. Conclusions

In summary, these results highlight the novel strategy of combining NBP plasma and para-coumaric acid to induce ferroptosis, a therapeutic approach that has not been previously proposed. This combination significantly enhances membrane permeability, increases RONS levels, and promotes lipid peroxidation, which accelerates the onset of ferroptosis in cancer cells. By inhibiting GPX4 and suppressing key regulatory pathways such as NRF2 and xCT, this strategy effectively overcomes common resistance mechanisms, particularly resistance to apoptosis. These findings are especially significant, as they provide new insights into the mechanistic pathways of cell death, positioning NBP plasma and para-coumaric acid-based treatment as promising and innovative therapeutic options. This approach represents a valuable first step toward advancing cancer therapies by selectively targeting cancer cells, offering an effective, noninvasive treatment that could complement the existing therapies. The successful induction of ferroptosis through co-treatment provides a strong foundation for future research and potential clinical applications.

## Figures and Tables

**Table 1:** List of primers utilized for this experiment.

Gene	Forward	Reverse
*GAPDH*	GAAGGTGAAGGTCGGAGTC	GAAGATGGTGATGGGATTTC
*LSH*	GATTTTGGATCGAATGCTGCCAG	ATGGACCCATCAAGCCTGCTGA
*SLC7A11*	TCCTGCTTTGGCTCCATGAACG	AGAGGAGTGTGCTTGCGGACAT
*GPX4*	ACAAGAACGGCTGCGTGGTGAA	GCCACACACTTGTGGAGCTAGA
*HMOX1*	CCAGCGGGCCAGCAACAAAGTGC	AAGCCTTCAGTGCCCACGGTAAGG
*NRF2*	TTCCCGGTCACATCGAGAG	TCCTGTTGCATACCGTCTAAACT
*KEAP1*	TGCTAACCTCTATACATGCAACT	GCAACGGTCAAAGAAGACT
*P53*	AGGAAATTTGCGTGTGGAGTAT	TCCGTCCCAGTAGATTACCACT
*PARP*	GCTCCCAGGAGTCAAGAGTG	TCAGGTCGTTCTGAGCCTTT
*Caspase3*	CATACTCCACAGCACCTGGTTA	ACTCAAATTCTGTTGCCACCTT
*BAX*	GAGAGGTCTTCCGAGTGG	GGAGGAAGTCCAATGTCCAG

## Data Availability

The data that support the findings of this study are available from the corresponding author upon reasonable request.

## Supplementary Materials

The following supporting information can be downloaded at https://www.mdpi.com/article/10.3390/biom15050691/s1. Figure S1: (A) Transition energy levels of nitrogen molecules. (B) Emission spectrum lines for N_2_ SPS (second positive system) and FPS (first positive system). (C) Plasma temperature and density for the plasma jet. Figure S2: Effect of combination treatment (para-coumaric acid with NBP-Jet plasma) on the cell viability including H460, A549, and MRC5 cells: The cell viability of (A) H460, (B) A549, and (C) MRC5 by using para-coumaric acid alone at several doses (0–100 µg/mL), which is incubated for 2 h. Combined effects of 12.5 µg/mL of para-coumaric acid and NBP-jet plasma exposure at various durations (15–300 s). Figure S3: Effect of combination treatment on cell morphology and movement: (A) The H460 and A549 cell morphologies were changed by para-coumaric acid and NBP-jet plasma treatment in the bright field after a 24 h incubation period. (B) Microscopic image of cell morphology after treatment by using crystal violet staining assay. (C) Cell migration assay of the H460 and A549 cells by using crystal violet staining. The incubation periods were 1 h and 12 h. Figure S4: (A) Intracellular RNS and ROS level of the H460 and A549 cells after 24 h of para-coumaric acid with NBP-jet plasma treatment. (B) The quantitative analysis of RNS and ROS level by using ImageJ. Figure S5: (A) Annexin V-FITC flow cytometric analysis displays the proportion of the total apoptotic cell population in both cell lines under different treatment conditions. (B) Quantitation rates of early and late apoptosis events for H460 and A549 cells. Figure S6: Generation and evaluation of lung adenocarcinoma spheroids in 24-well plates: (A) Inhibition of spheroid growth formation of the H460 and A549 cell lines in bright field after para-coumaric acid with NBP-jet plasma treatment during 1 h and 12 h incubation period. (B) Quantification of the H460 cell spheroid size. (C) Quantification of the A549 cell spheroid size. (D) Quantification of the expression level of p-p38 in H460 and (E) quantification of the expression level of p-p38 in A549 cells. Figure S7: The qRT-PCR analysis for DNA damage and cell death markers, including p53, PARP, Caspase-3, and Bax, in lung cancer cells treated with para-coumaric acid and NBP-jet plasma: (A) p53, (B) PARP, (C) Caspase-3, and (D) Bax in the H460 cells, and (E) p53, (F) PARP, (G) Caspase-3, and (H) Bax in the A549 cells. The GAPDH gene was used for data normalization. Figure S8: (A) Intracellular chelatable iron level in the H460 and A549 cells after 24 h of para-coumaric acid and NBP-jet plasma treatment. (B) The quantitative analysis of chelatable iron level in both cell lines by using ImageJ. (C) The chelatable iron level of the H460 and A549 cells by using flow cytometry analysis with Phen Green SK for detecting iron level kit. (D) Quantification of flow cytometry intensity in both cell lines using ImageJ software. Figure S9: Labile iron pool (LIP) level in (A) H460, and (B) A549 cells after 24 h of para coumaric acid and NBP-jet plasma treatment. File S1: Western Blotting Figures.
