# Supplementary material for: Enhancing Ferroptosis in Lung Adenocarcinoma Cells via the Synergistic Action of Nonthermal Biocompatible Plasma and a Bioactive Phenolic Compound
Source: Biomolecules. 2025 May 9;15(5):691. doi: 10.3390/biom15050691 (PMC12108647; doi:10.3390/biom15050691)
Supplement: Supplementary file 1 [file biomolecules-15-00691-s001.zip › biomolecules-3583984-File S1.Western Blotting Figures.pdf]

Enhancing ferroptosis in lung adenocarcinoma cells via the synergistic action of nonthermal biocompatible plasma and a bioactive phenolic compound

Sabnaj Khanam[1,2], Young June Hong[2,3], Youngsun Kim[4], Eun Ha Choi [1,2,5 *], Ihn Han [1,2 *]

[1] Department of Plasma Bio-Display, Kwangwoon University, Seoul 01897, Korea

[2] Plasma Bioscience Research Center, Applied Plasma Medicine Center, Kwangwoon University, Seoul 01897, Korea

[3] Advanced technology research institute, Nayuda Co., Seoul 04067, Korea

[4] Department of Obstetrics and Gynecology, Kyung Hee University Medical Center, Seoul, 02447, Korea

[5] Department of Electronic and Biological Physics, Kwangwoon University, Seoul 01897, Korea

*Correspondence to Ihn Han and Eun Ha Choi

Dasanje 301, Kwangwoon ro 20, Nowongu, Seoul, Korea of Republic

email: hanihn@kw.ac.kr, phone: 082-2-940-5666, Fax: 082-2-940-5664

Replicate -3

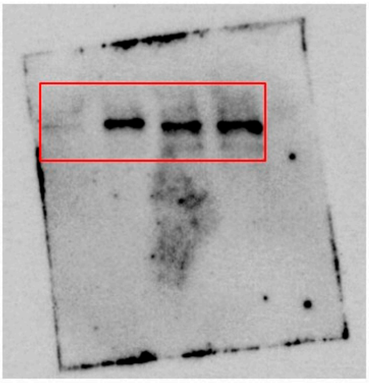

P53/H460

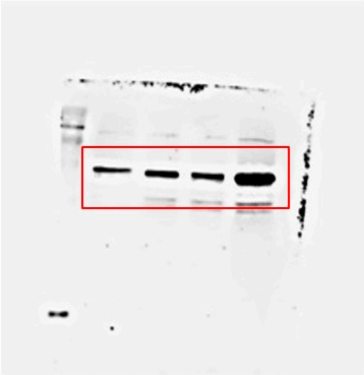

P53/A549

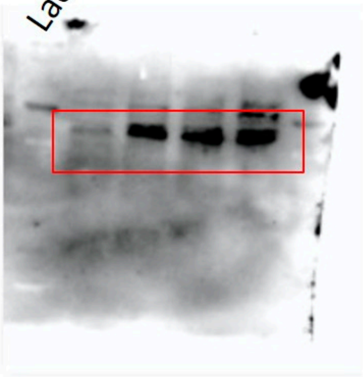

Cleaved PARP/H460

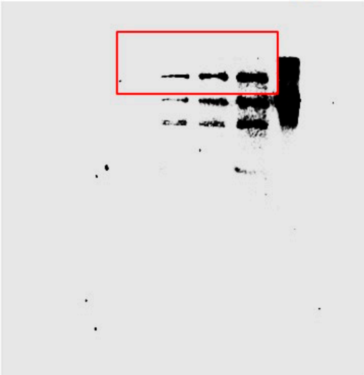

Cleaved PARP/A549

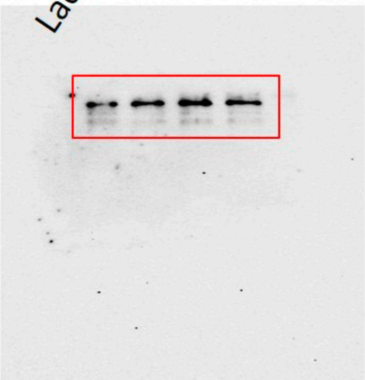

PARP/H460

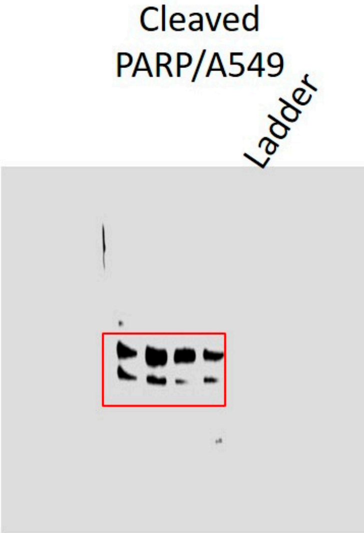

PARP/A549

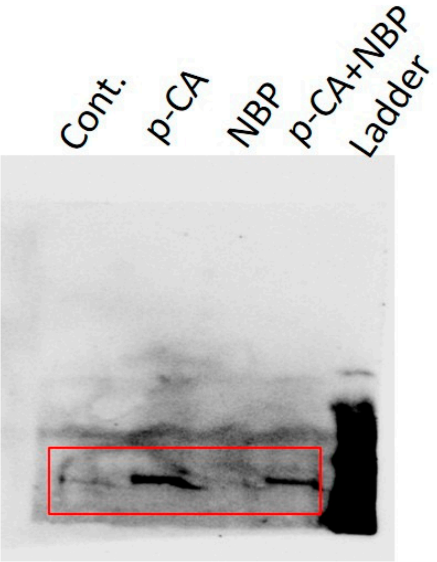

Cleaved Caspase-3
/H460

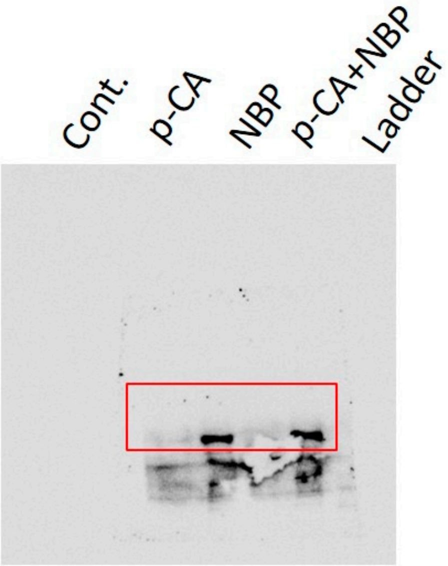

Cleaved Caspase-3
/A549

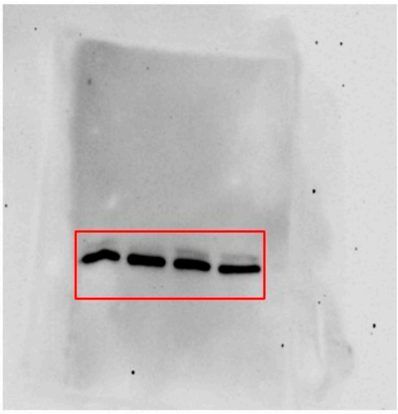

Caspase-3 /H460

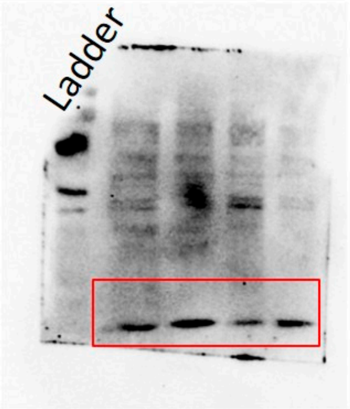

Caspase-3 /A549

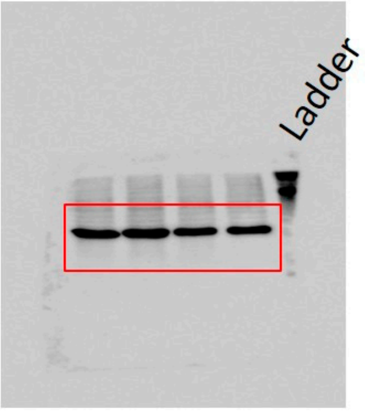

GAPDH /H460

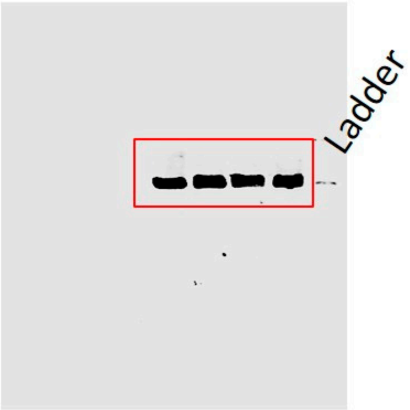

GAPDH /A549

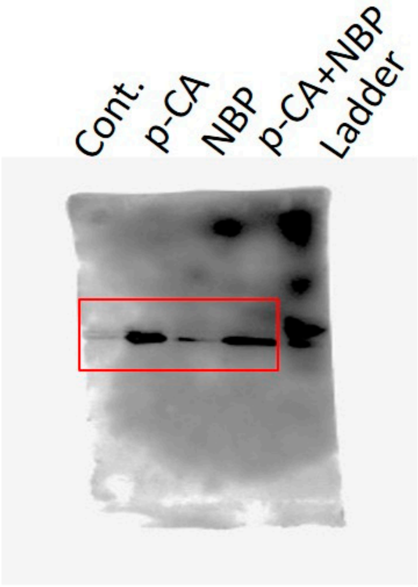

P-ERK /H460

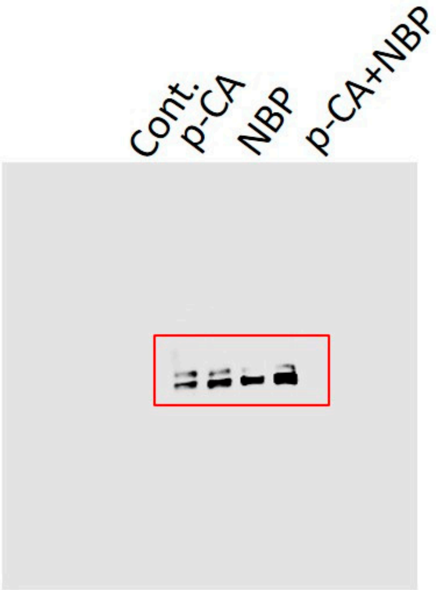

P-ERK /A549

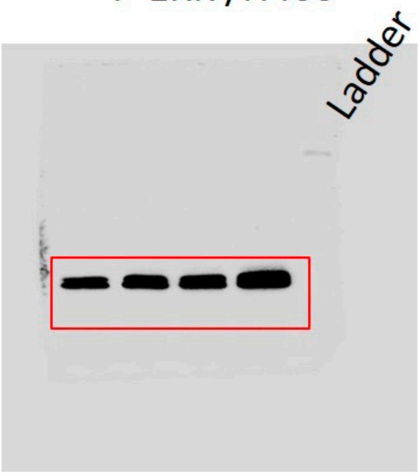

ERK /H460

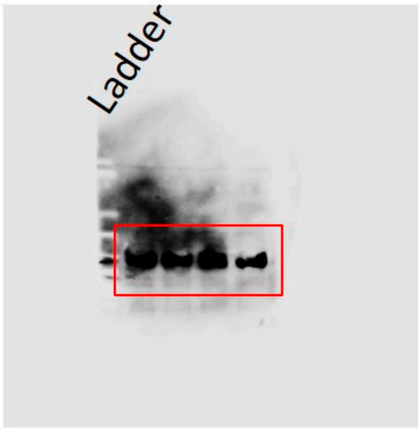

ERK /A549

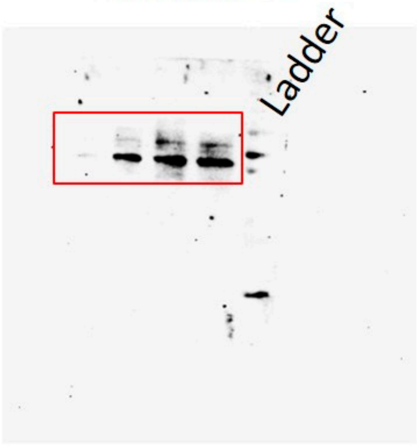

P-JNK /H460

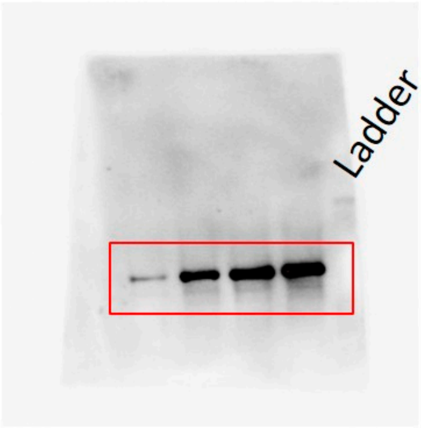

P-JNK /A549

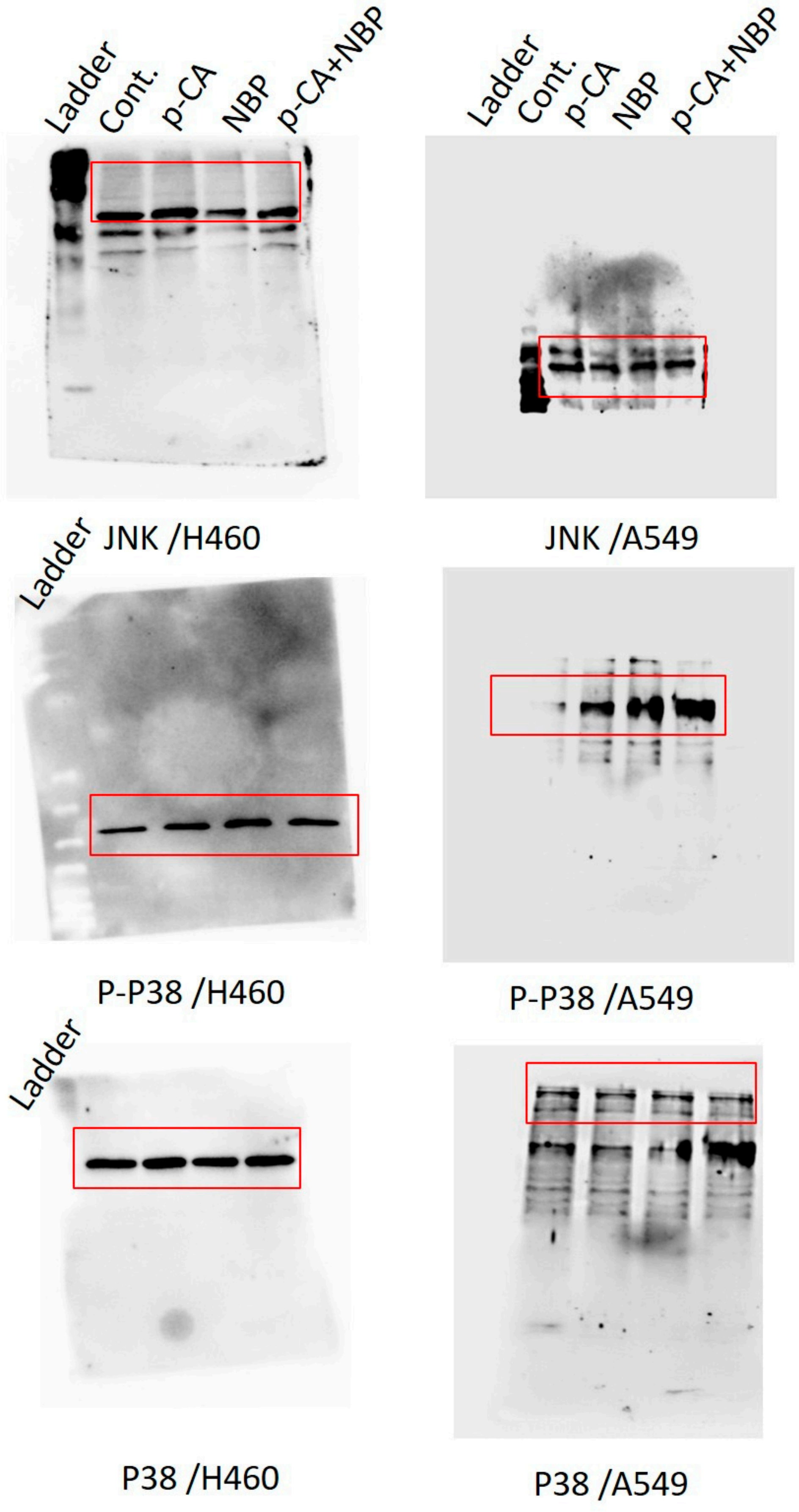
Ladder
Cont.
p-CA
NBP
p-CA+NBP
Ladder
Cont.
p-CA
NBP
p-CA+NBP
JNK /H460
JNK /A549
Ladder
P-P38 /H460
P-P38 /A549
Ladder
P38 /H460
P38 /A549

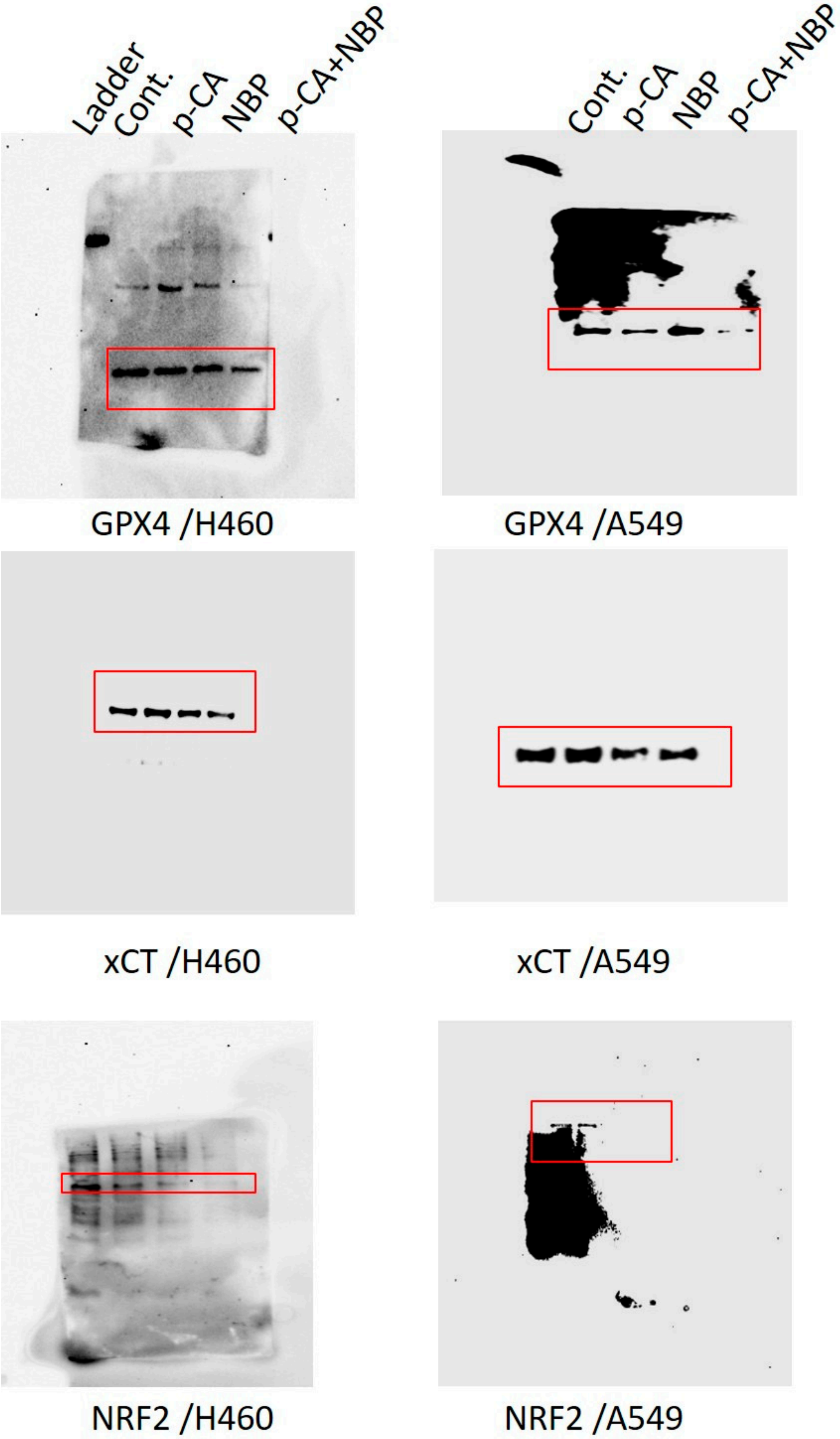
Ladder
Cont.
p-CA
NBP
p-CA+NBP
Cont.
p-CA
NBP
p-CA+NBP
GPX4 /H460
GPX4 /A549
xCT /H460
xCT /A549
NRF2 /H460
NRF2 /A549

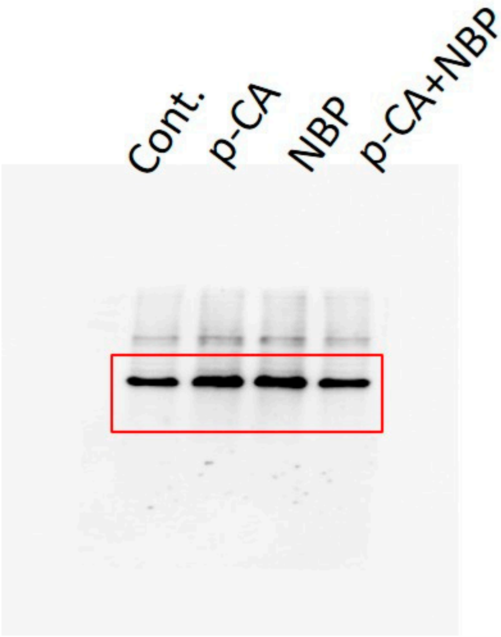

GAPDH /H460

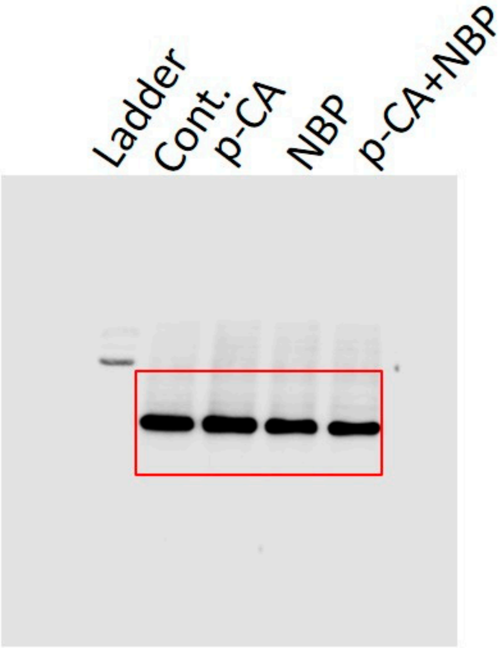

GAPDH /A549

Replicate -2

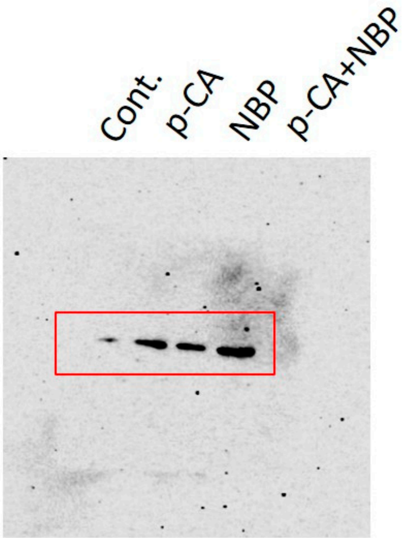

P53 /H460

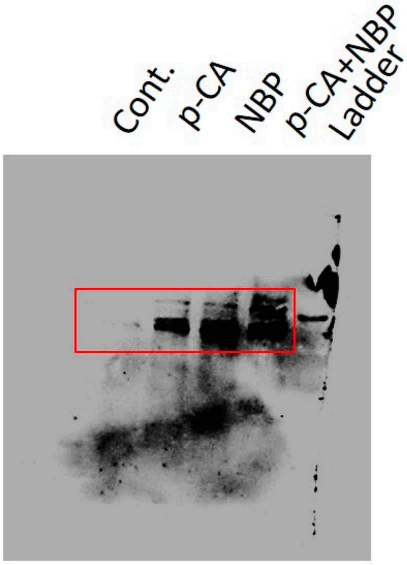

P53 /A549

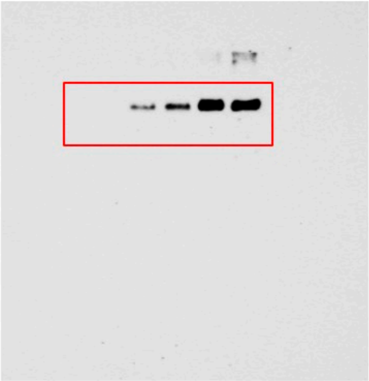

Cleaved PARP /H460

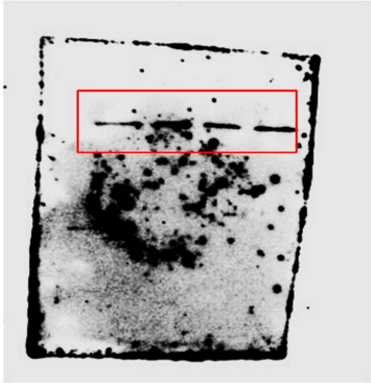

Cleaved PARP /A549

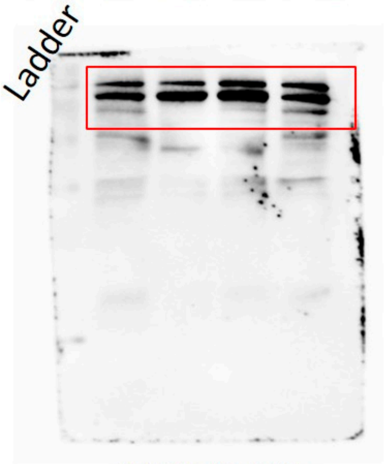

PARP /H460

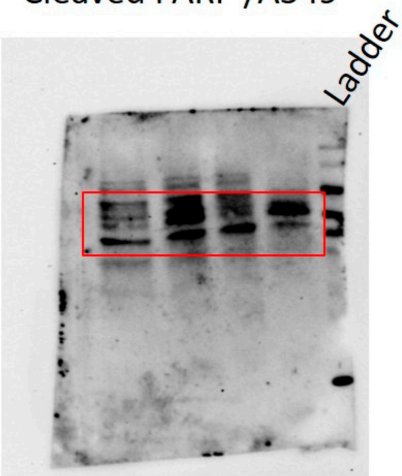

PARP /A549

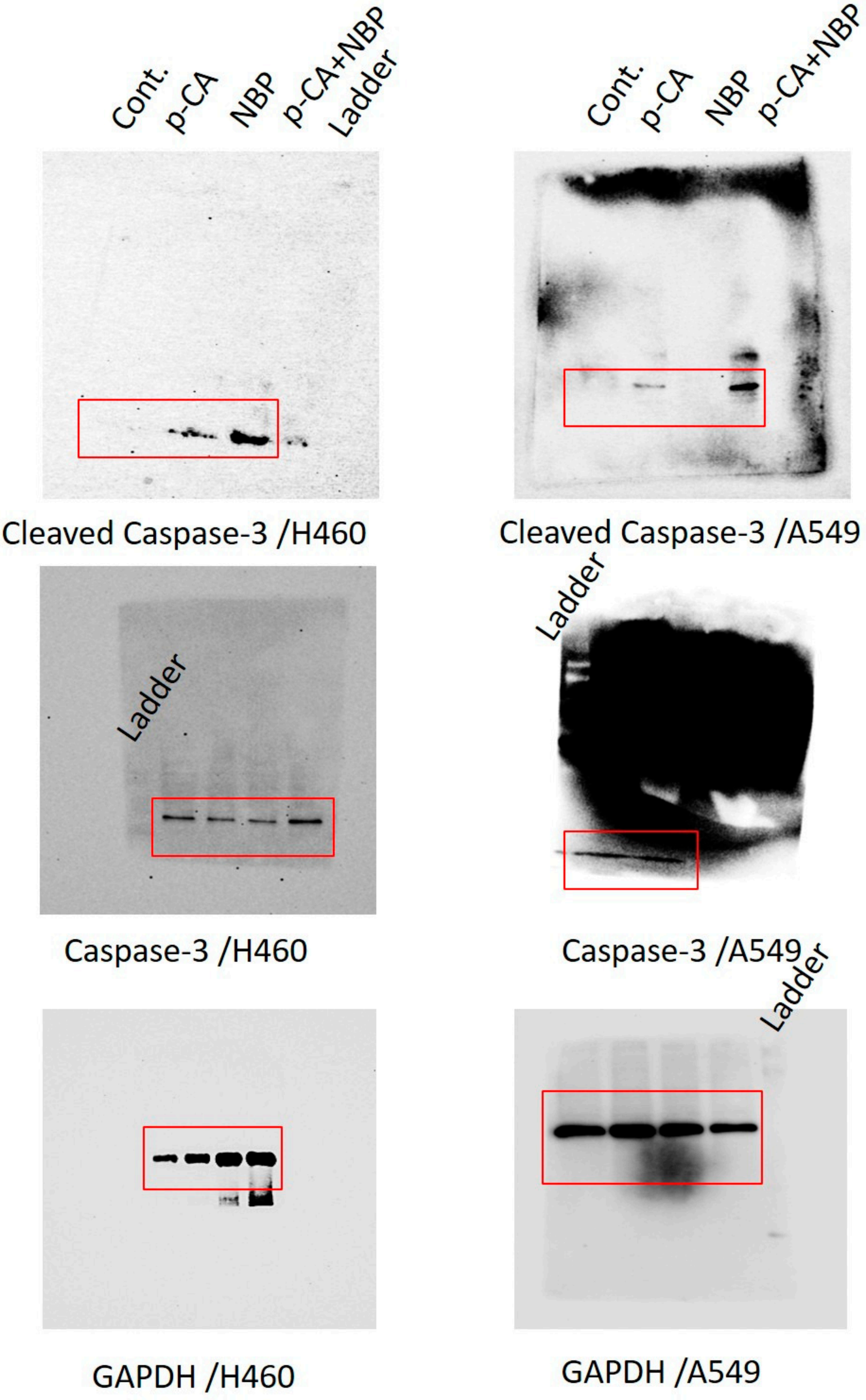
Cont.
p-CA
NBP
p-CA+NBP
Ladder
Cleaved Caspase-3 /H460
Cont.
p-CA
NBP
p-CA+NBP
Cleaved Caspase-3 /A549
Ladder
Caspase-3 /H460
Ladder
Caspase-3 /A549
Ladder
GAPDH /H460
GAPDH /A549

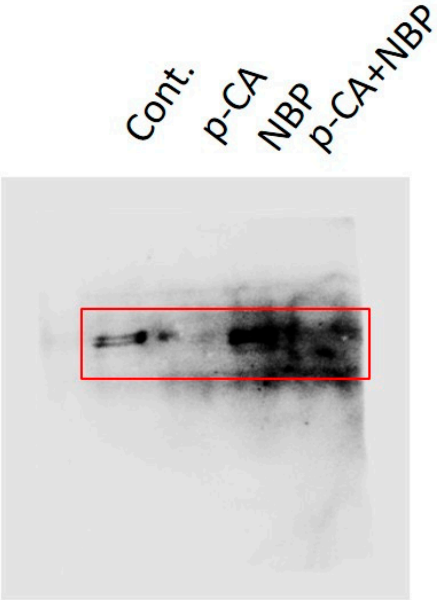

P-ERK /H460

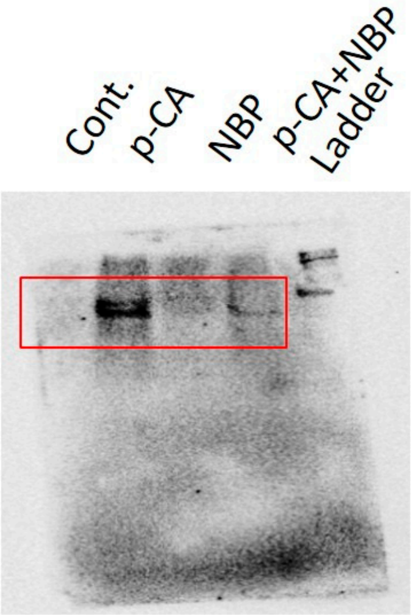

P-ERK/A549

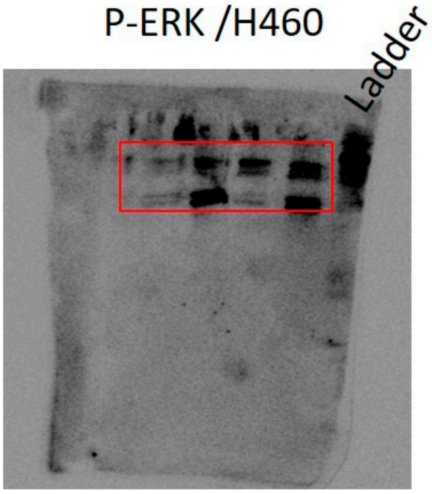

ERK/H460

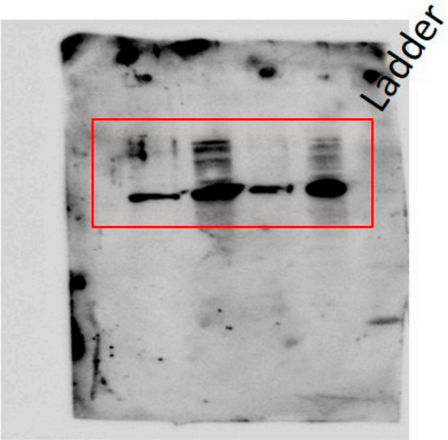

ERK/A549

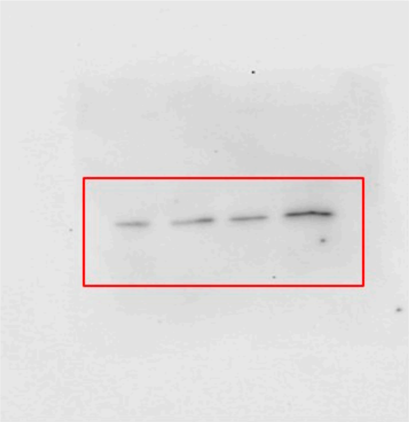

P-JNK/H460

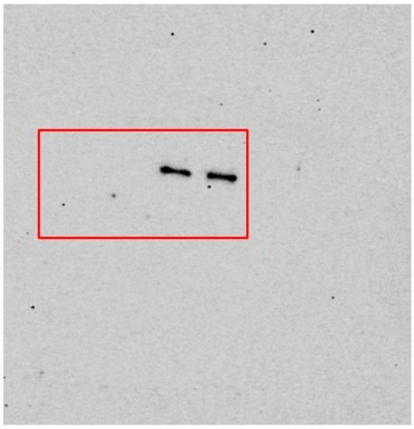

P-JNK/A549

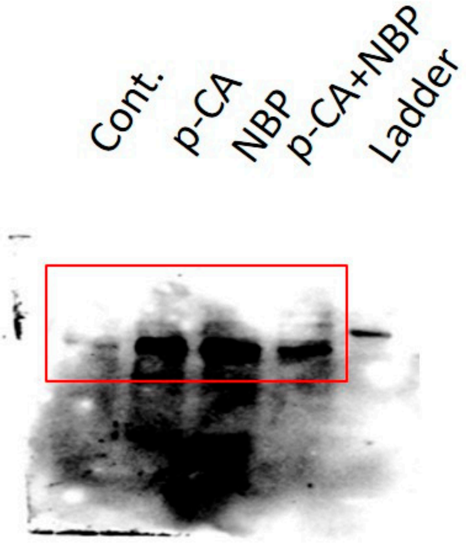

JNK/H460

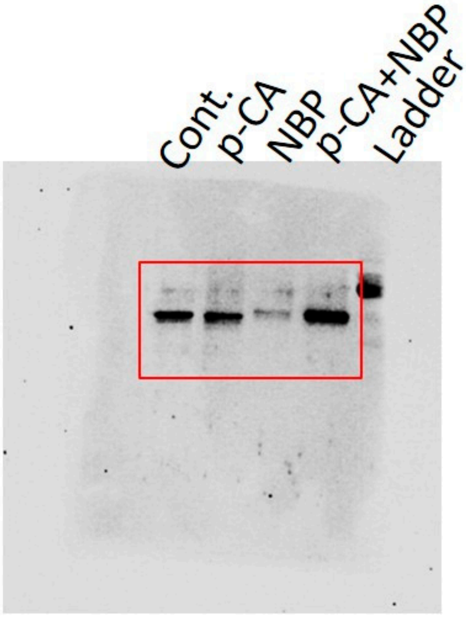

JNK/A549

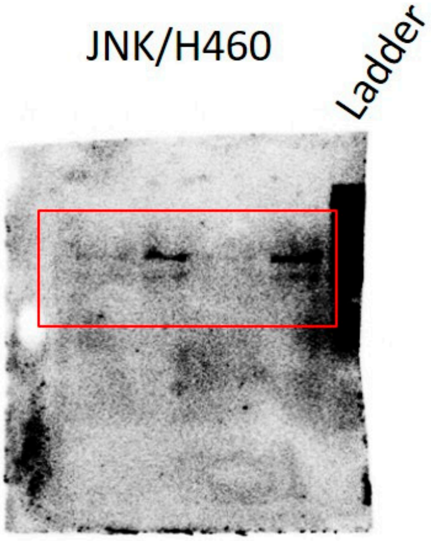

P-P38/H460

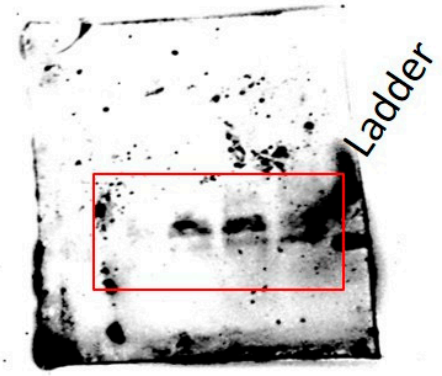

P-P38/A549

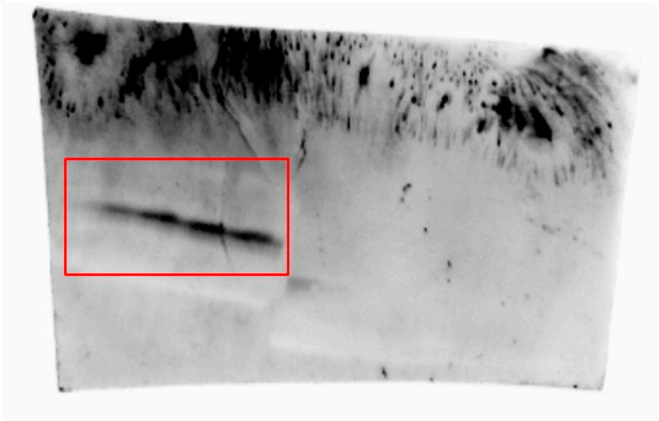

P38/H460

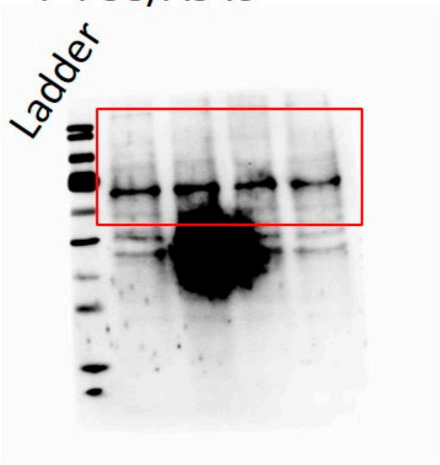

P38/A549

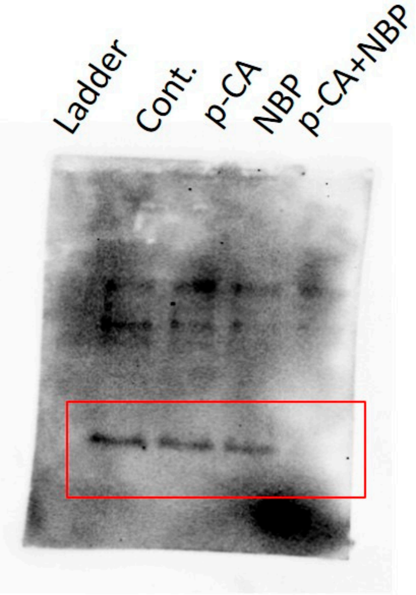

GPX4/H460

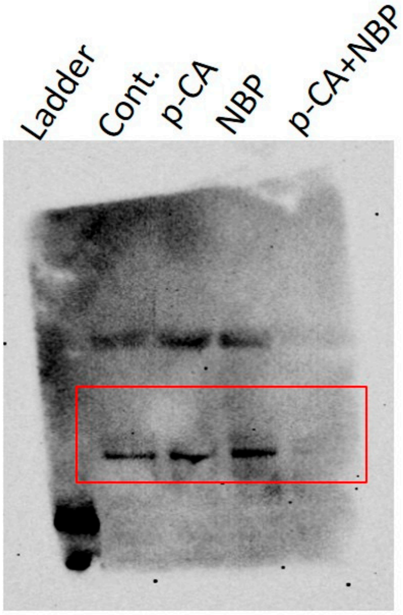

GPX4/A549

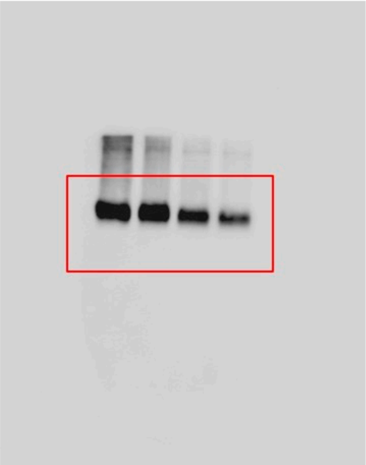

xCT/H460

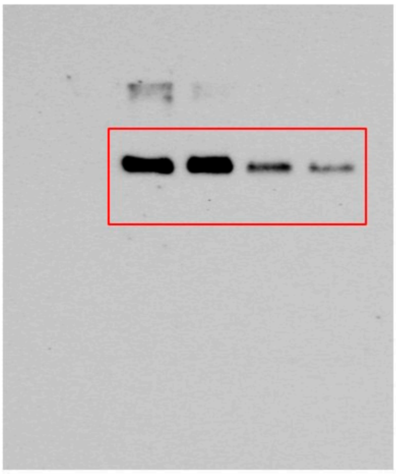

xCT/A549

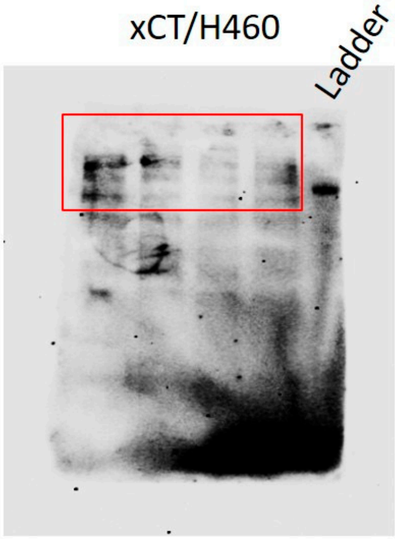

NRF2/H460

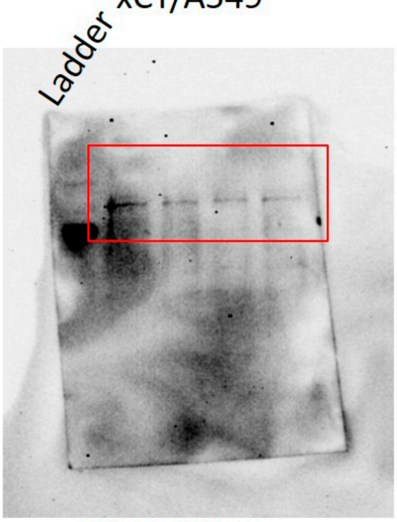

NRF2/A549

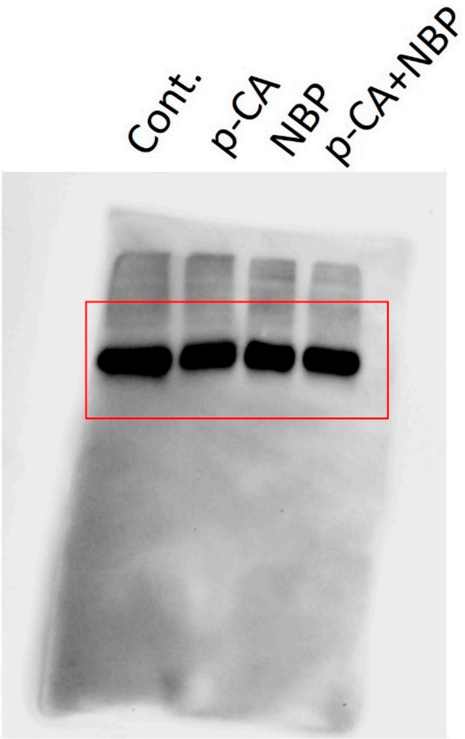

GAPDH/H460

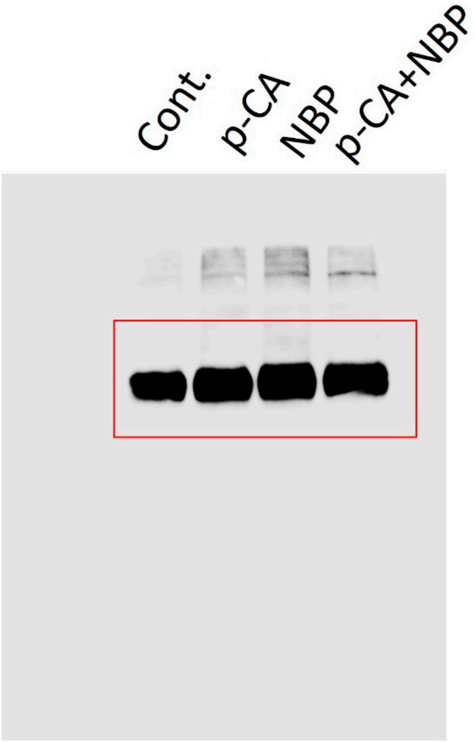

GAPDH/A549

Replicate -1

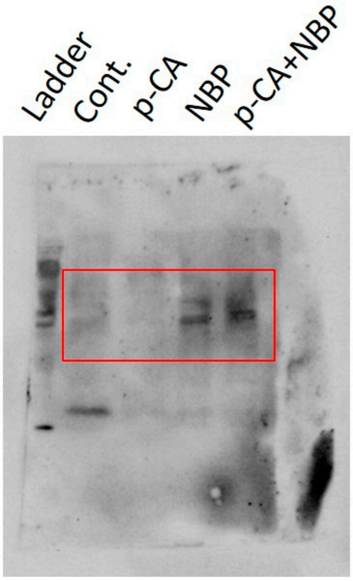

P53/H460

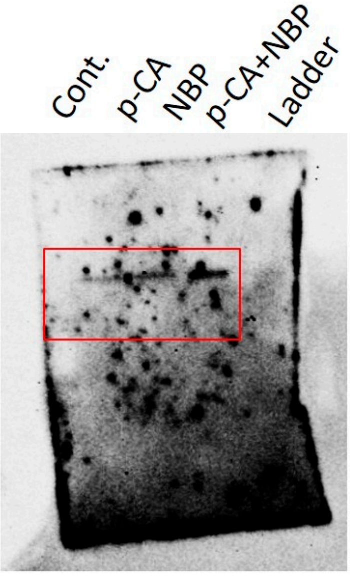

P53/A549

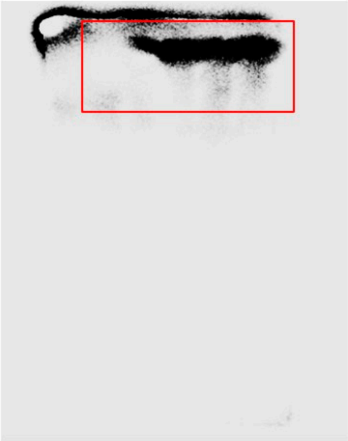

Cleaved PARP/H460

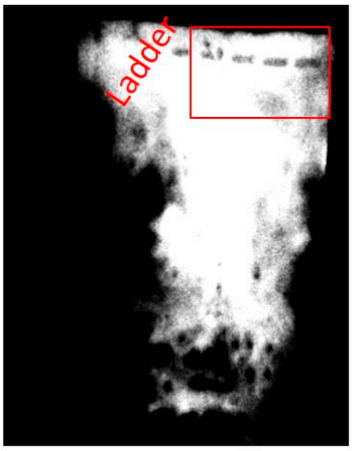

Cleaved PARP/A549

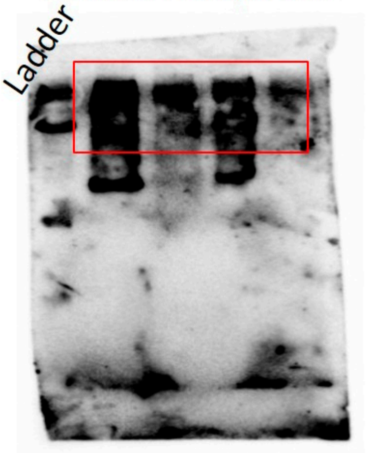

PARP/H460

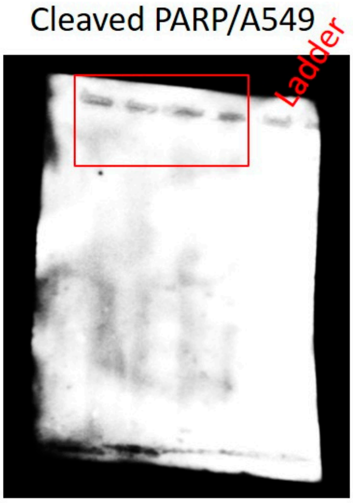

PARP/A549

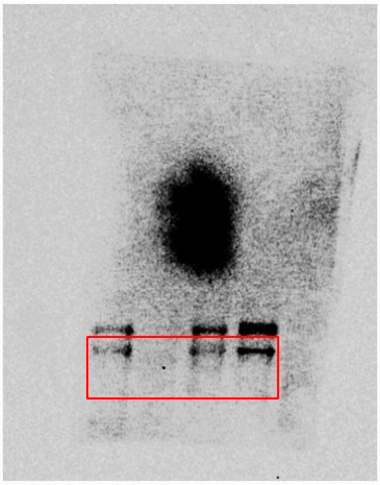

Cleaved Caspase-3/H460

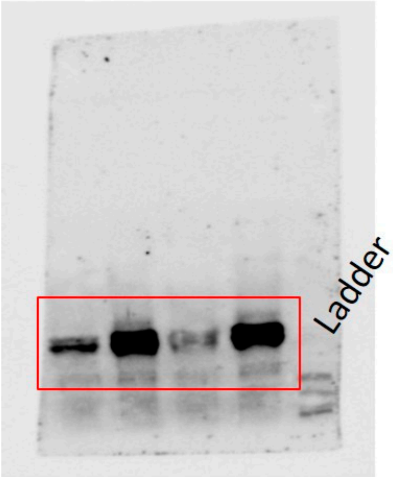

Cleaved Caspase-3/A549

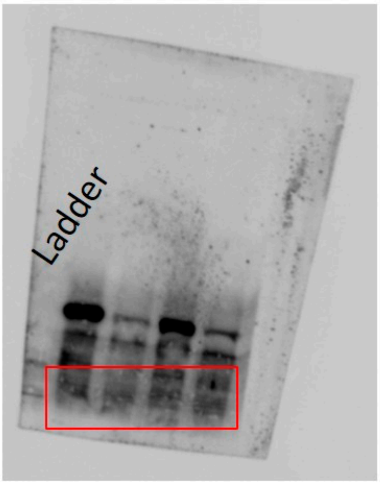

Caspase-3/H460

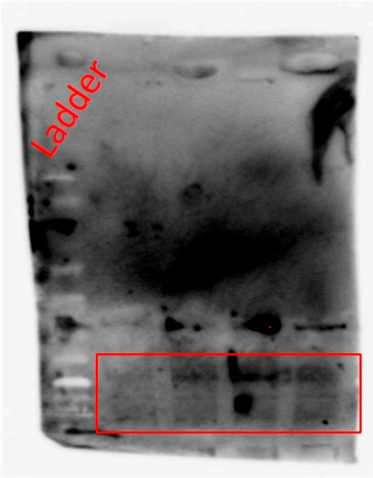

Caspase-3/A549

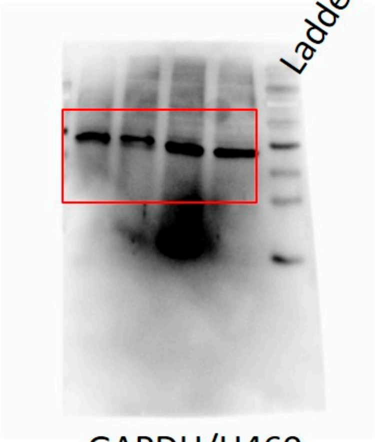

GAPDH/H460

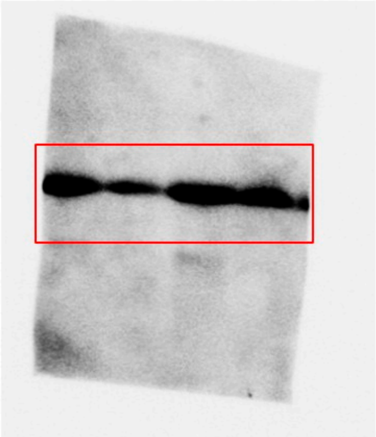

GAPDH/A549

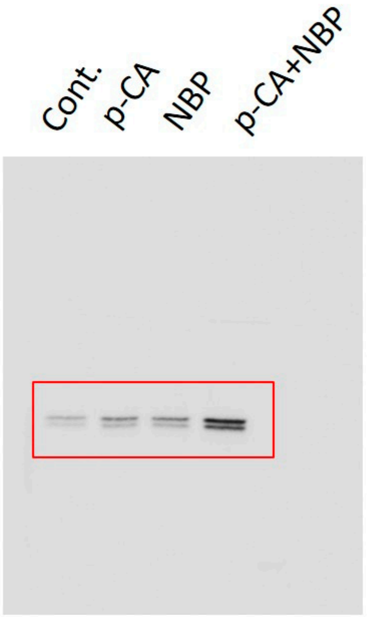

P-ERK/H460

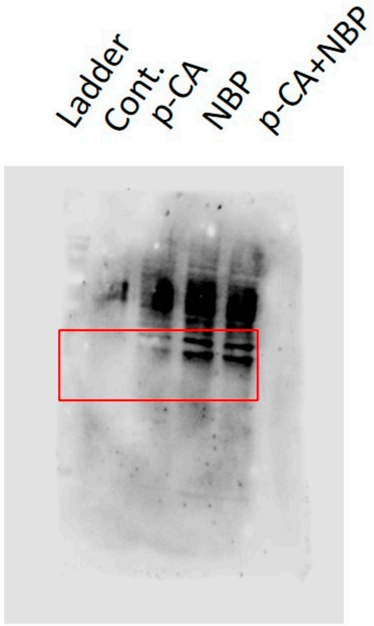

P-ERK/A549

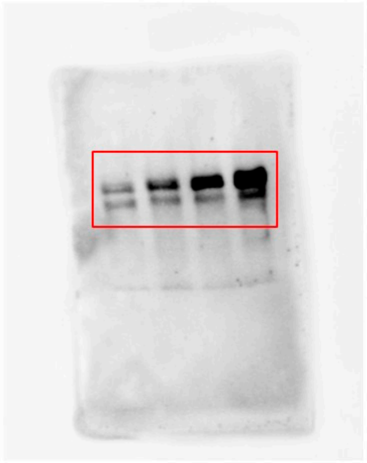

ERK/H460

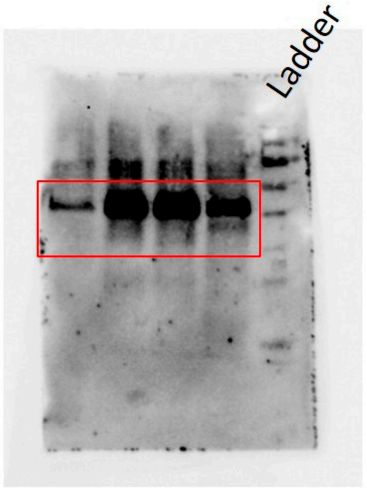

ERK/A549

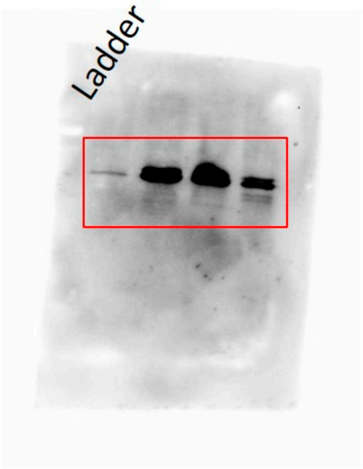

P-JNK/H460

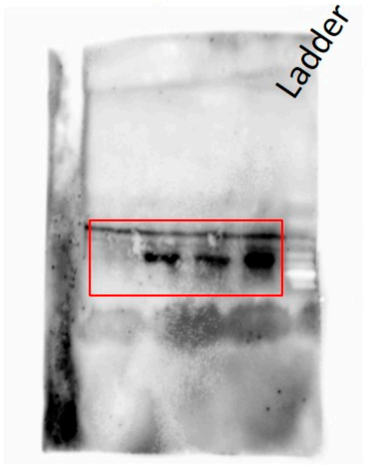

P-JNK/A549

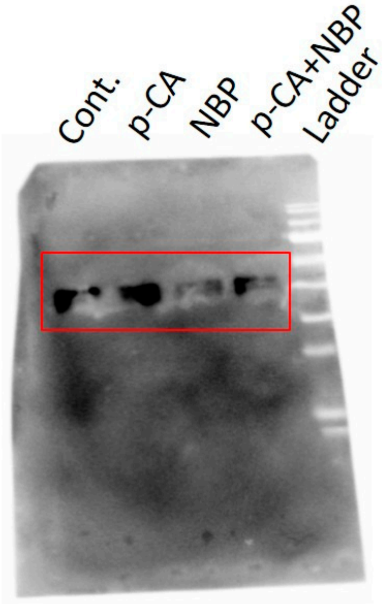

JNK/H460

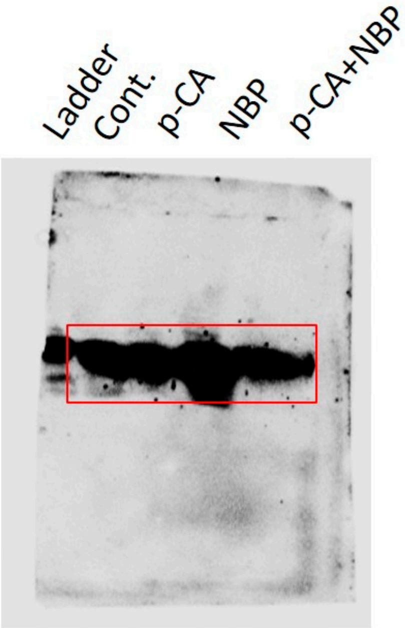

JNK/A549

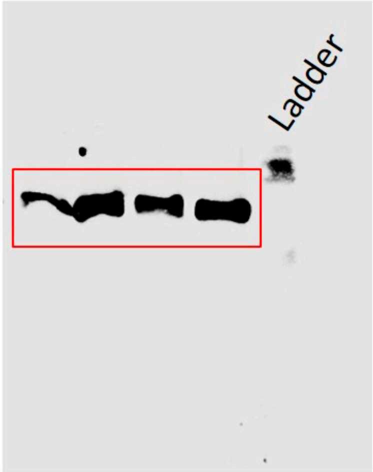

P-P38/H460

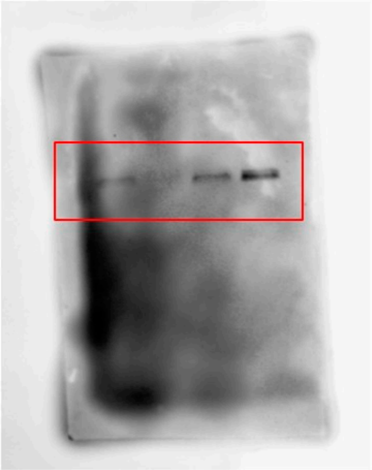

P-P38/A549

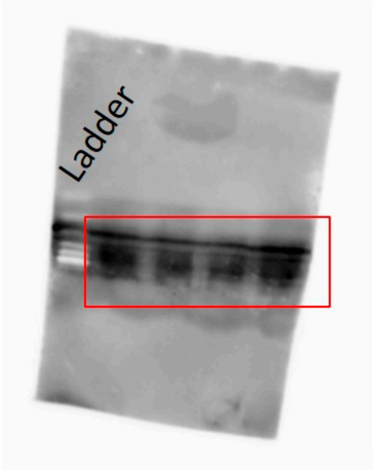

P38/H460

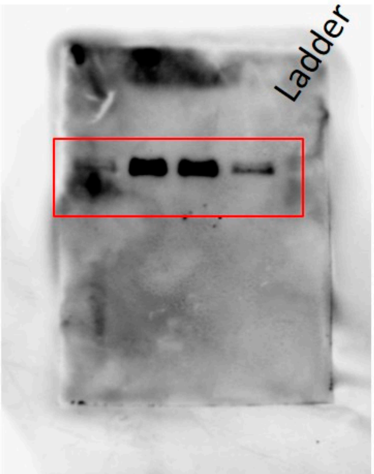

P38/A549

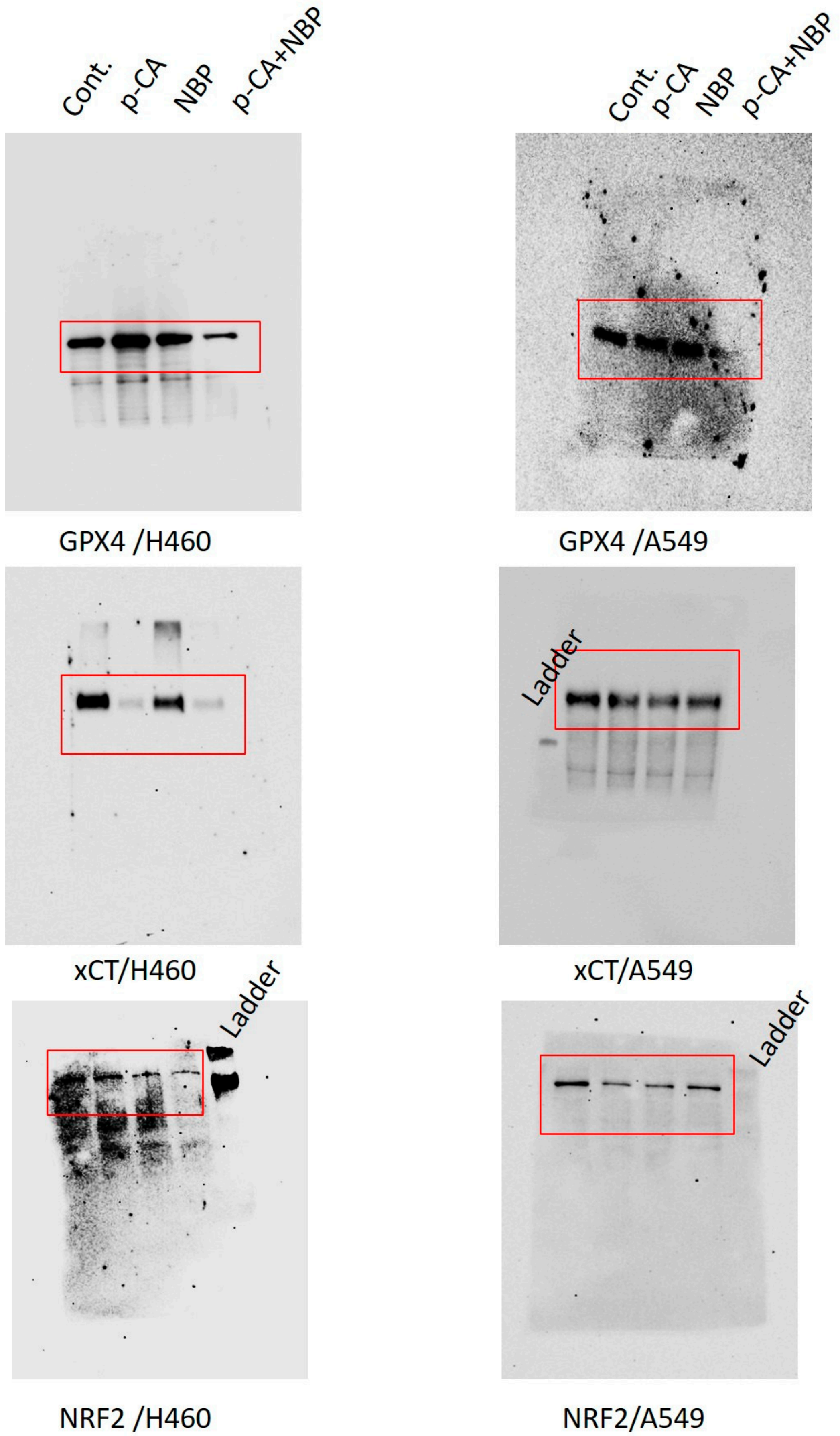
Cont.
p-CA
NBP
p-CA+NBP
Cont.
p-CA
NBP
p-CA+NBP
GPX4 /H460
GPX4 /A549
Ladder
xCT/H460
xCT/A549
Ladder
Ladder
NRF2 /H460
NRF2/A549

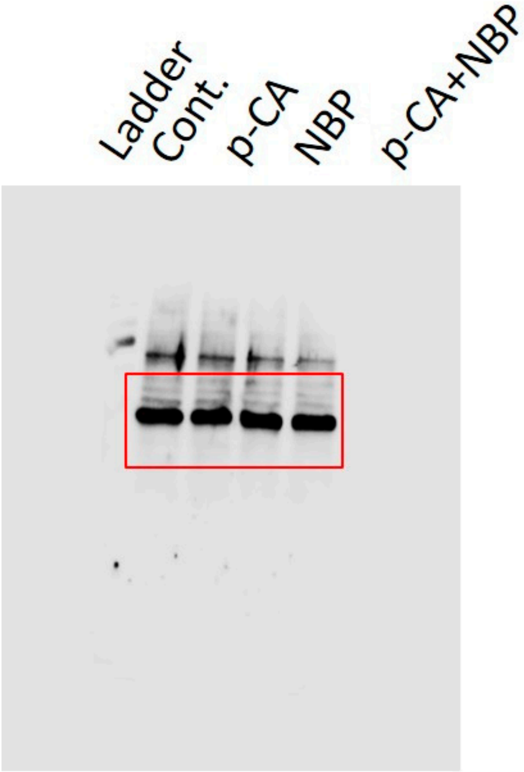

GAPDH /H460

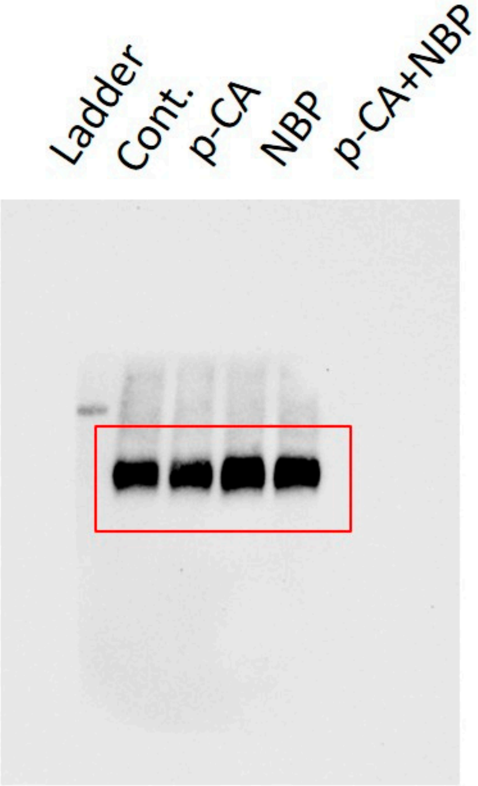

GAPDH /A549
