# Supplementary material for: Enhancing Ferroptosis in Lung Adenocarcinoma Cells via the Synergistic Action of Nonthermal Biocompatible Plasma and a Bioactive Phenolic Compound
Source: Biomolecules. 2025 May 9;15(5):691. doi: 10.3390/biom15050691 (PMC12108647; doi:10.3390/biom15050691)
Supplement: Supplementary file 1 [file biomolecules-15-00691-s001.zip › biomolecules-3583984-supplementary.pdf]

Supplementary Material for

Enhancing ferroptosis in lung adenocarcinoma cells via the synergistic action of nonthermal biocompatible plasma and a bioactive phenolic compound

Sabnaj Khanam[1,2], Young June Hong[2,3], Youngsun Kim[4], Eun Ha Choi [1,2,5] *, Ihn Han [1,2] *

[1] Department of Plasma Bio-Display, Kwangwoon University, Seoul 01897, Korea

[2]Plasma Bioscience Research Center, Applied Plasma Medicine Center, Kwangwoon University, Seoul 01897, Korea

[3] Advanced technology research institute, Nayuda Co., Seoul 04067, Korea

[4] Department of Obstetrics and Gynecology, Kyung Hee University Medical Center, Seoul, 02447, Korea

[5] Department of Electronic and Biological Physics, Kwangwoon University, Seoul 01897, Korea

*Correspondence to Ihn Han and Eun Ha Choi

Dasanje 301, Kwangwoon ro 20, Nowongu, Seoul, Korea of Republic

email: hanihn@kw.ac.kr, phone: 082-2-940-5666, Fax: 082-2-940-5664

Result

S1. Measurement of the plasma temperature and density

To analyze air plasma, we calculated the plasma temperature and density using a nitrogen molecule collisional radiative model [1–3]. The nitrogen plasma generated in ambient air can be transitioned from the excited states *A* $^3\Sigma_u^+$, *B* $^3\Pi_g$, *C* $^3\Pi_u$ and the ground state *X* $^1\Sigma_g^+$ due to the electron impact excitation effect of plasma [1–3]. We analyzed the nitrogen spectra of the NBP-jet to measure the respective plasma temperatures and densities. Figure S1 shows the transition energy levels of the nitrogen molecule and measured spectrum of the air plasma. Figure S1A showed the energy-level diagram for the transition of nitrogen molecules. During plasma discharge, electron-impact excitation causes transitions from the ground state X level to the excited states of the upper A, B, and C levels. This is shown in the spectrum in Figure S1B, which consists of the N_2 second positive system (SPS) for the C-B transition and the N_2 first positive system (FPS) for the B-A transition. Experimentally, in the air plasma, the intensity of the N_2 SPS was significantly higher than that of the N_2 FPS.

For the electron impact excitation of the N_2 plasma, the following reactions can be occurred: [1–3]

$$e + N_2(X) \rightarrow e + N_2(A) \quad \text{(S1)}$$

$$e + N_2(X) \rightarrow e + N_2(B) \quad \text{(S2)}$$

$e + N_2(X) \rightarrow e + N_2(C)$ (S3)

For the excited state transition processes [1–3]:

$N_2(B) \rightarrow N_2(A) + hv$ (S4)

$N_2(C) \rightarrow N_2(B) + hv$ (S5)

For the collisional excitation and deactivation processes [1–3]:

$N_2(B) + N_2(X) \rightarrow N_2(A) + N_2(X)$ (S6)

$N_2(A) + N_2(X, 5 \leq v \leq 14) \rightarrow N_2(B) + N_2(X)$ (S7)

$N_2(A) + N_2(A) \rightarrow N_2(B) + N_2(X, v = 0)$ (S8)

$N_2(A) + N_2(A) \rightarrow N_2(C) + N_2(X, v = 0)$ (S9)

For the wall deactivation processes [1–3]:

$N_2(A) \rightarrow N_2(X)$; wall deactivation (S10)

Nitrogen molecules in lower states $A\ ^3\Sigma_u^+$ and $B\ ^3\Pi_g$ have the effect of spontaneous emission in higher states $B\ ^3\Pi_g$ and $C\ ^3\Pi_u$ and emit spectra of N_2 FPS (first positive system) and N_2 SPS (second positive system) [1–3]. Also, the excited molecule of $A\ ^3\Sigma_u^+$ state can have the wall deactivation [1–3]. For practical applications of atmospheric plasma, it is also important to consider the energy pooling reaction, which involves the transition to the $B\ ^3\Pi_g$, $C\ ^3\Pi_u$, and $X\ ^1\Sigma_g^+$ states due to the collision between two molecules in the $A\ ^3\Sigma_u^+$ state [1–3]. The total balance equations for three excited $A\ ^3\Sigma_u^+$, $B\ ^3\Pi_g$, and $C\ ^3\Pi_u$ state can be made [1–3].

In the case of $N_2\,(A)$,

$$2n_A^2(k_{AAB} + k_{AAC}) + (k_{wall} + k_{AX}n_v)n_A - (A_B + k_{BX}n_g)n_B - n_e n_g Q_A = 0 \quad \text{(S11)}$$

In the case of $N_2\,(B)$,

$$n_A^2 k_{AAB} + k_{AX}n_v n_A - (A_B + k_{BX}n_g)n_B + A_C n_C + n_e n_g Q_B = 0 \quad \text{(S12)}$$

In the case of $N_2\,(C)$,

$$n_A^2 k_{AAC} - A_C n_C + n_e n_g Q_C = 0 \quad \text{(S13)}$$

The n_e, n_A, n_B, and n_C are the density of electron and excited states $A\ ^3\Sigma_u^+$, $B\ ^3\Pi_g$, and $C\ ^3\Pi_u$, respectively. n_v is the neutral gas density for the vibrational temperature, and the n_g corresponds to the neutral gas density associated with the rotational temperature. The vibrational and rotational temperatures were determined from the intensity of the spectra of the second positive system using the Boltzmann plot method [4,5]. The vibrational temperature can be estimated using the Boltzmann plot method for each peak intensity in N_2 SPS [4,5]. The rotational temperature was obtained by applying a Boltzmann plot to the decreasing intensity around the selected wavelength of 380.49 nm [4,5]. The k_{AX} and k_{BX} are the rate coefficients for collision processes between $A\ ^3\Sigma_u^+$ - $X\ ^1\Sigma_g^+$ and $B\ ^3\Pi_g$ - $X\ ^1\Sigma_g^+$ states, respectively [1]. The k_{AAB} and k_{AAC} are the rate coefficients for the process of exiting to upper states $B\ ^3\Pi_g$ and $C\ ^3\Pi_u$ after the collision between excited states $A\ ^3\Sigma_u^+$ [1]. k_{wall} is the rate coefficient of wall deactivation, which is obtained using the diffusion model with a coefficient of wall reflection [1]. The Q_A, Q_B, and Q_C are the

rate coefficients for electron impact excitation from the ground state to excited states $A\ ^3\Sigma_u^+$, $B\ ^3\Pi_g$, and $C\ ^3\Pi_u$, respectively [1,6]. The A_B and A_C are the transition probabilities of excited states $B\ ^3\Pi_g$ and $C\ ^3\Pi_u$ [1]. We can establish three second-order simultaneous equations for the three unknown variables corresponding to the excited molecule densities n_A, n_B, and n_C as shown in equations (S11)-(S13). These variables can be easily solved using Python's mathematical libraries. Additionally, the excited molecule densities for arbitrary electron temperatures

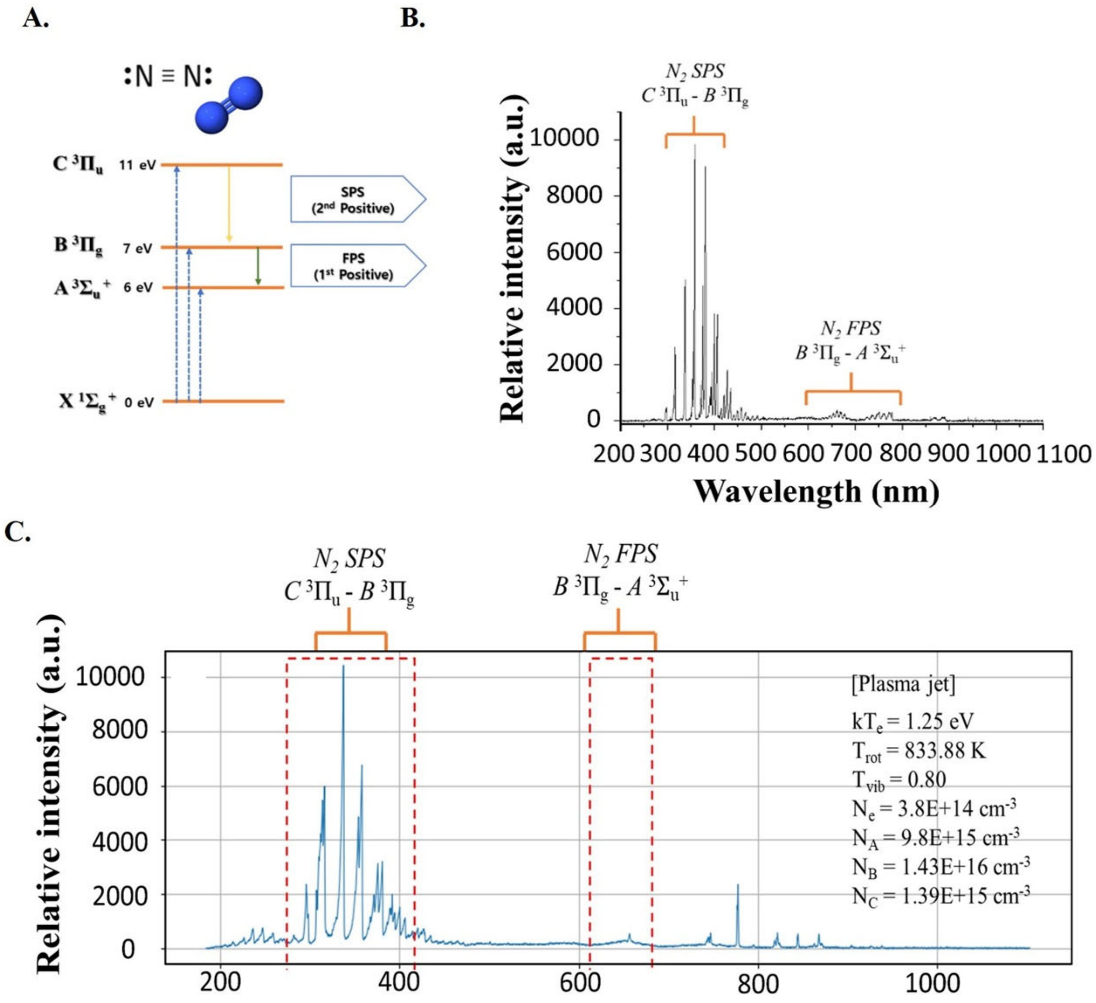

Figure S1. (A) Transition energy levels of nitrogen molecules. (B) Emission spectrum lines for N_2 SPS (second positive system) and FPS (first positive system). (C) Plasma temperature and density for the plasma jet.

and densities can also be determined using equations, (S11) – (S13). The ratio of molecular densities for nitrogen SPS and FPS can be expressed as follows: [1]

$$\mathrm{R}(kT_e, n_e) = \frac{A_C n_C}{A_B n_B} \tag{S14}$$

The estimated values shown in equation, (S14) were compared as the ratio of the measured emission lines to the ratio of SPS density to N_2 FPS density, which depends on the electron temperature and density. The selected N_2 SPS emissions were from 12 lines with wavelengths of 295.32, 313.60, 315.93, 337.13, 353.67, 357.69, 371.05, 375.54, 380.49, 389.46, 399.84, and 405.94 nm [7]. The N_2 FPS line is one line at a wavelength of 654.49 nm [7]. The R-values in equation. (S4), which depend on arbitrary electron temperature and density, were determined for the 12 lines of N_2 SPS and the one line of FPS in atmospheric-pressure air plasma. The specific electron temperature can be obtained by comparing the measured emission line ratio in the OES data of the air plasma. These electron temperatures can be expressed in relation to arbitrary electron densities and are obtained for each of the 12 line ratios [4,5]. We determined a specific electron density at the same electron temperature [4,5]. Using the method described in references [4] and [5], we obtained the plasma temperature and density by solving equations (S11)–(S13) and analyzing the nitrogen emission spectrum, which included the intensities of the N_2 SPS and FPS. Plasma temperatures include three parameters like electron, rotational, and vibrational temperatures. Plasma densities include electron, and excited A, B, and C state densities of the N_2 molecules. The plasma temperatures and densities of the NBP plasma jet were shown in Figure S1C. The NBP-jet uses a needle electrode, subjected to a significantly high electric field, resulting in a localized strong plasma generation region, where the spectral lines were observed to calculate the plasma temperature and density.

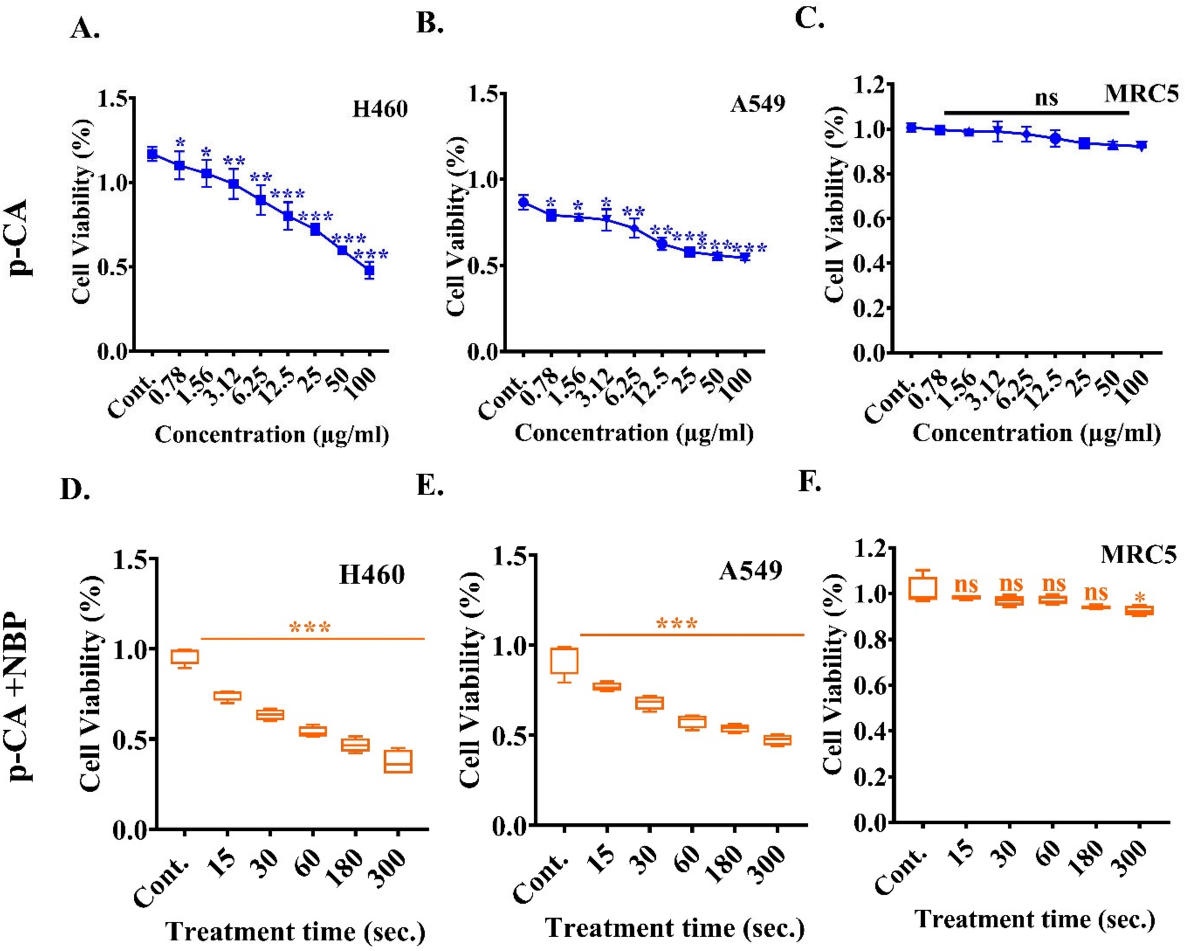

Figure S2. Effect of combination treatment (para coumaric acid with NBP-Jet plasma) on the cell viability including, H460, A549, and MRC5 cells. The cell viability of (A) H460, (B) A549, and (C) MRC5 by using para coumaric acid alone at several doses (0-100µg/ml), which is incubated for 2 hours. Combined effects of 12.5 µg/ml of para coumaric acid and NBP-jet plasma exposure at various durations (15 sec-300 sec). After giving co-treatment, cells were incubated for 24 hours. The data are presented as the means ± standard deviations of three independent experiments. Significance levels are denoted as *P < 0.05, **P < 0.01, and ***P < 0. 001 vs the control/treated group. ns, not significant.

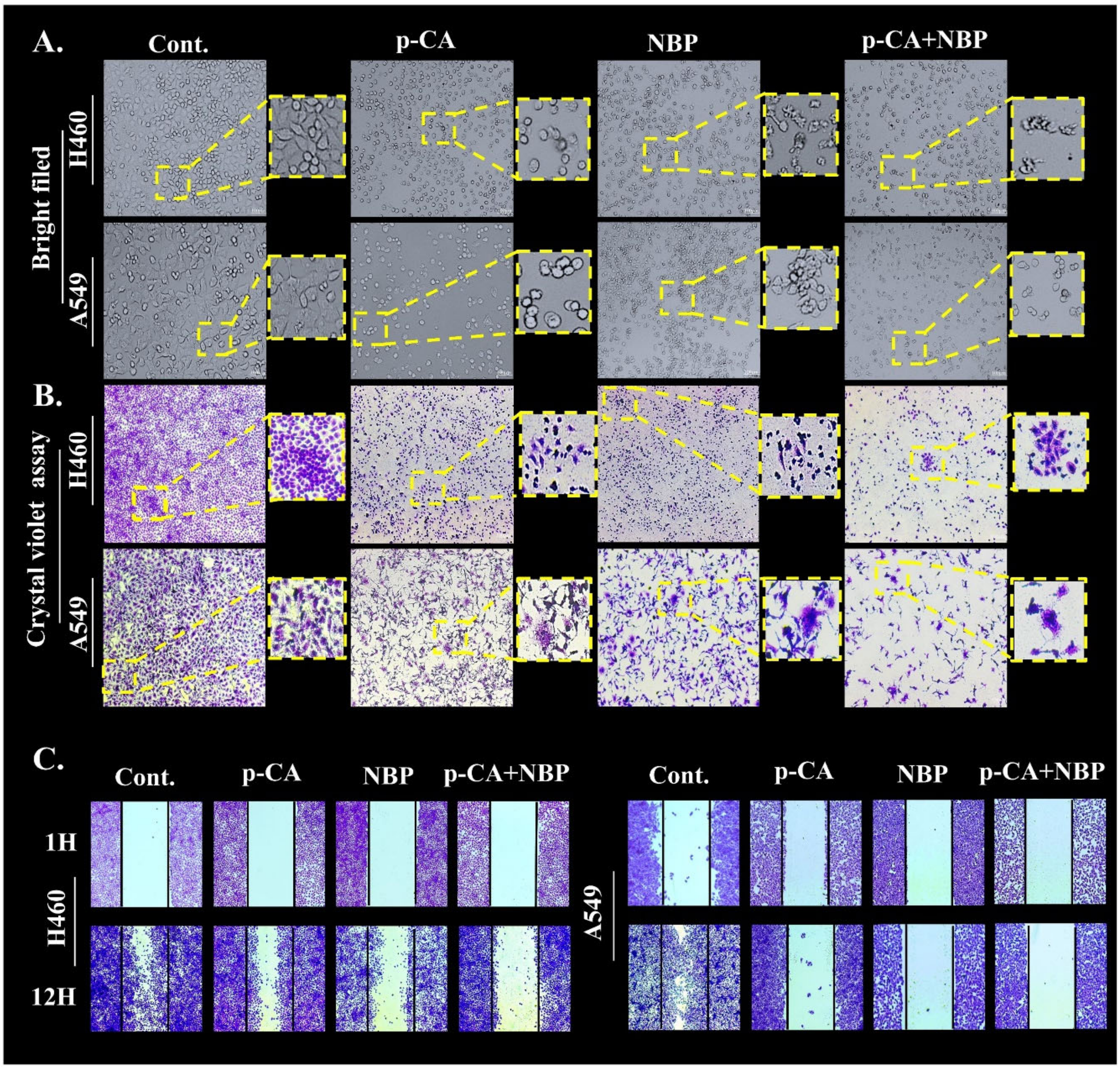

Figure S3. Effect of combination treatment on cell morphology and movement. (A) The H460 and A549 cells morphology were changed by para coumaric acid and NBP-jet plasma treatment in bright field after 24 hours incubation period. (B) Microscopic image of cell morphology after treatment by using crystal violet staining assay. (C) Cell movement assay of H460 and A549 cells by using crystal violet staining. The incubation periods were 1 h and 12 h. The photographs were taken in different wells, not the same well. Cell migration assay also performed in bright filed but that data is not presented here. Scale bar = 100 μm.

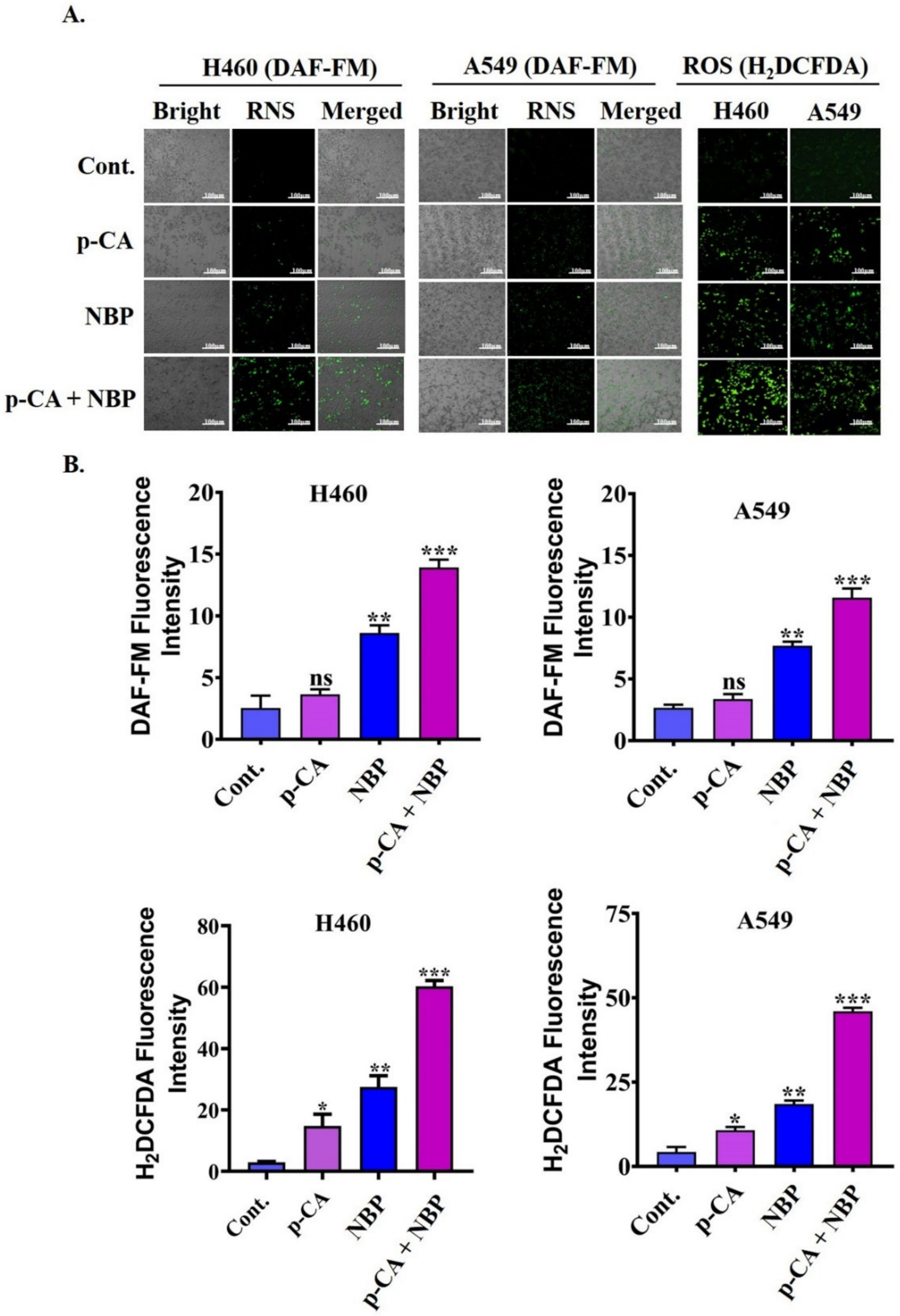

Figure S4. (A) Intracellular RNS and ROS level of H460 and A549 cells after 24 hours of para coumaric acid with NBP-jet plasma treatment. (B) the quantitative analysis of RNS and ROS level by using ImageJ. The data are presented as the means ± standard deviations of three independent experiments. Significance levels are denoted as *P < 0.05, **P < 0.01, and ***P < 0. 001 vs the control/treated group. ns, not significant.

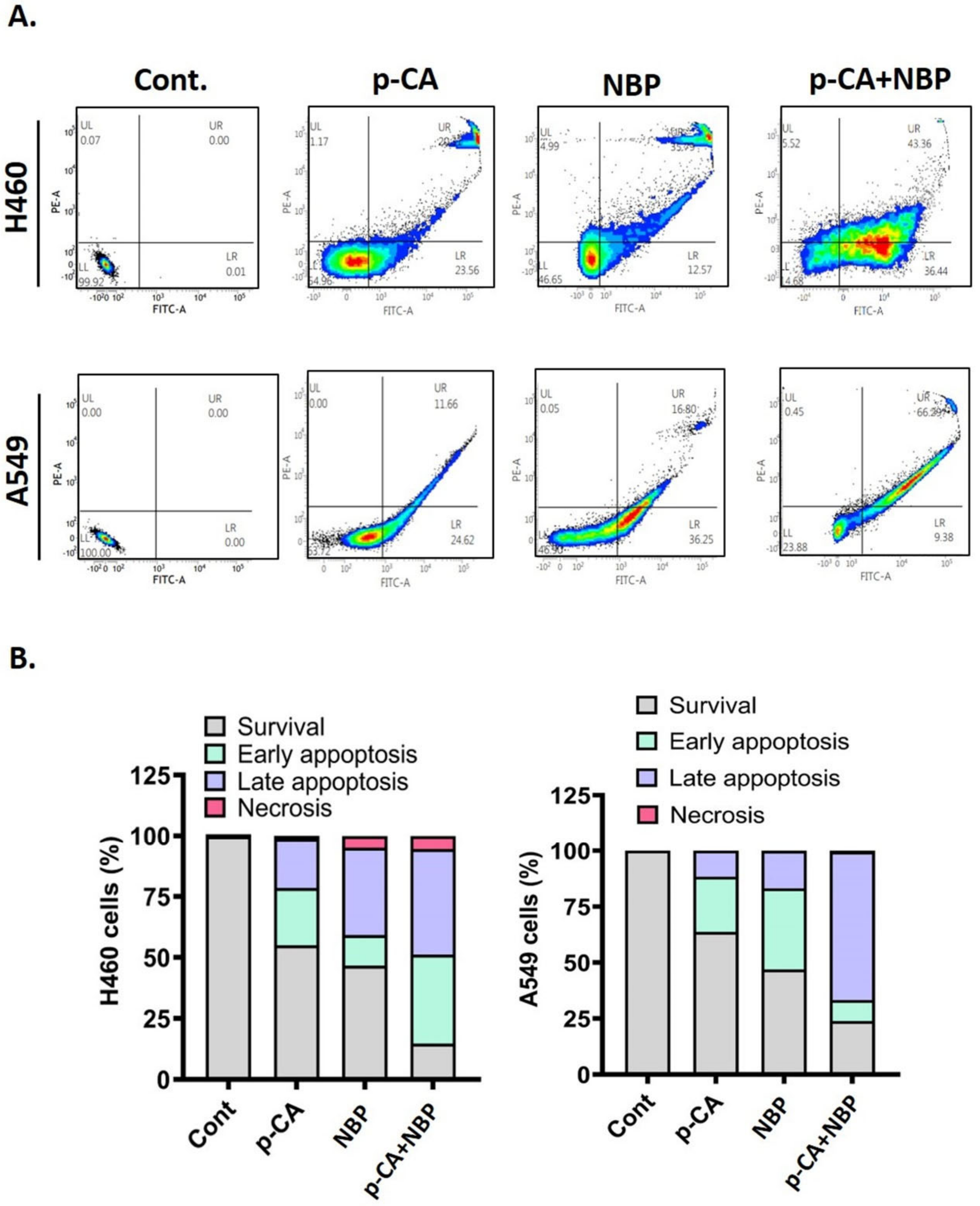

Figure S5. (A) Annexin V-FITC flow cytometric analysis displays the proportion of the total apoptotic cell population in both cell lines under different treatment conditions. (B) Quantitation rates of early and late apoptosis events for H460 and A549 cells.

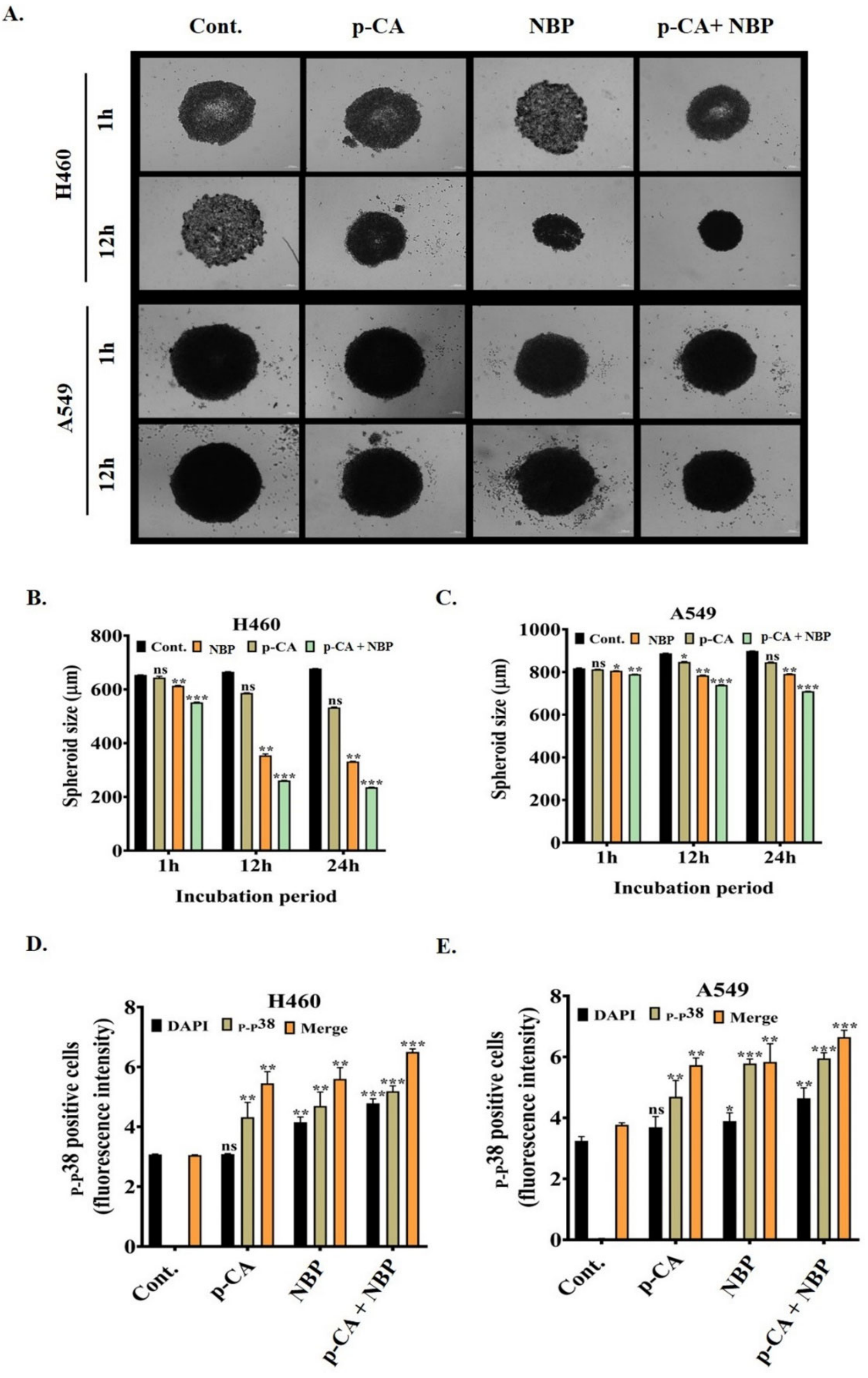

Figure S6. Generation and evaluation of lung adenocarcinoma spheroids in 24-well plates. (A) Inhibition of spheroid growth formation of H460 and A549 cell lines in bright field after para coumaric

acid with NBP-jet plasma treatment during 1h and 12h incubation period. (B) Quantification of H460 cells spheroid size. (C) Quantification of A549 cells spheroid size. (D) Quantification the expression level of p-p38 in H460 and (E) Quantification the expression level of p-p38 in A549 cells. The ImageJ software was used for quantification. The scale bar was 100 μm. The data are presented as the means ± standard deviations of three independent experiments. Significance levels are denoted as *P < 0.05, **P < 0.01, and ***P < 0. 001 vs the control/treated group. ns, not significant.

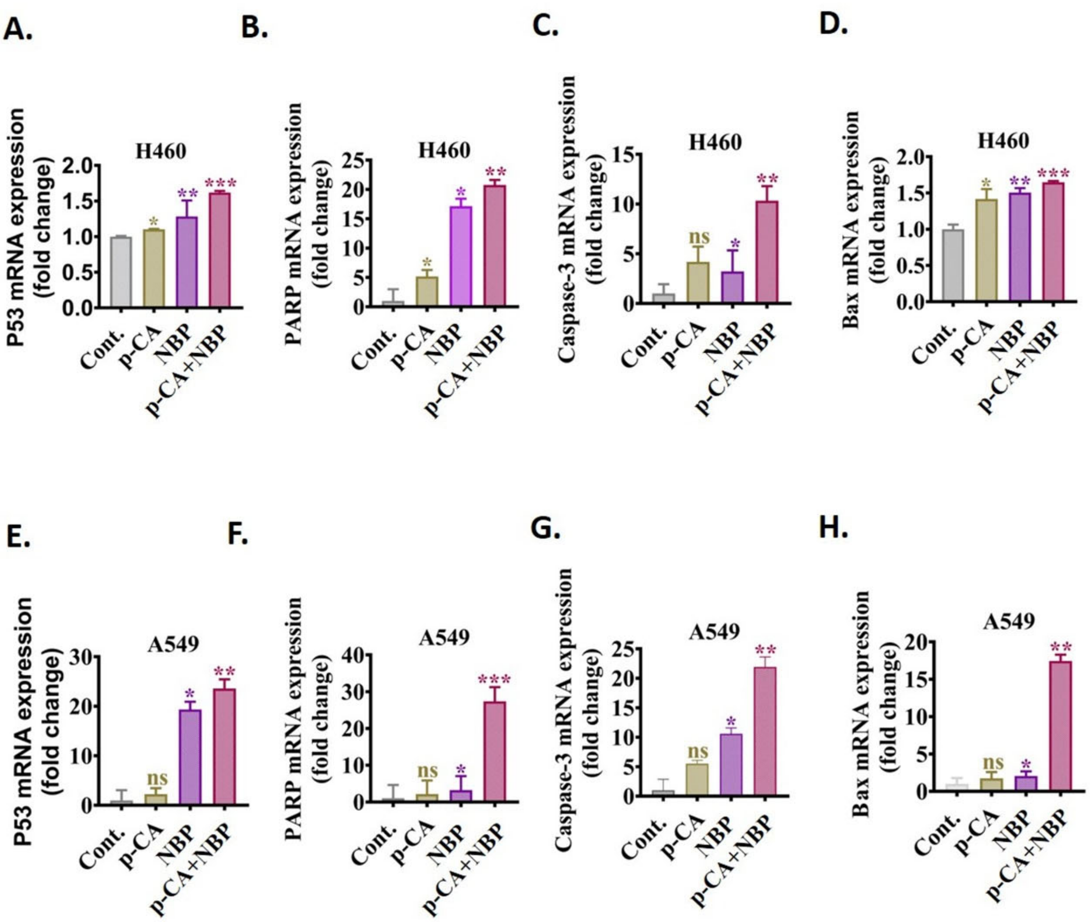

Figure S7. The qRT-PCR analysis for DNA damage and cell death markers, including p53, PARP, Caspase-3, and Bax, in lung cancer cells treated with para coumaric acid and NBP-jet plasma. (A) p53, (B) PARP, (C) Caspase-3, (D) Bax in H460 cells, (E) p53, (F) PARP, (G) Caspase-3, (H) Bax, in A549 cells. The GAPDH gene was used for data normalization. The data are presented as the means ± standard deviations of three independent experiments. Significance levels are denoted as *P < 0.05, **P < 0.01, and ***P < 0. 001 vs the control/treated group. ns, not significant.

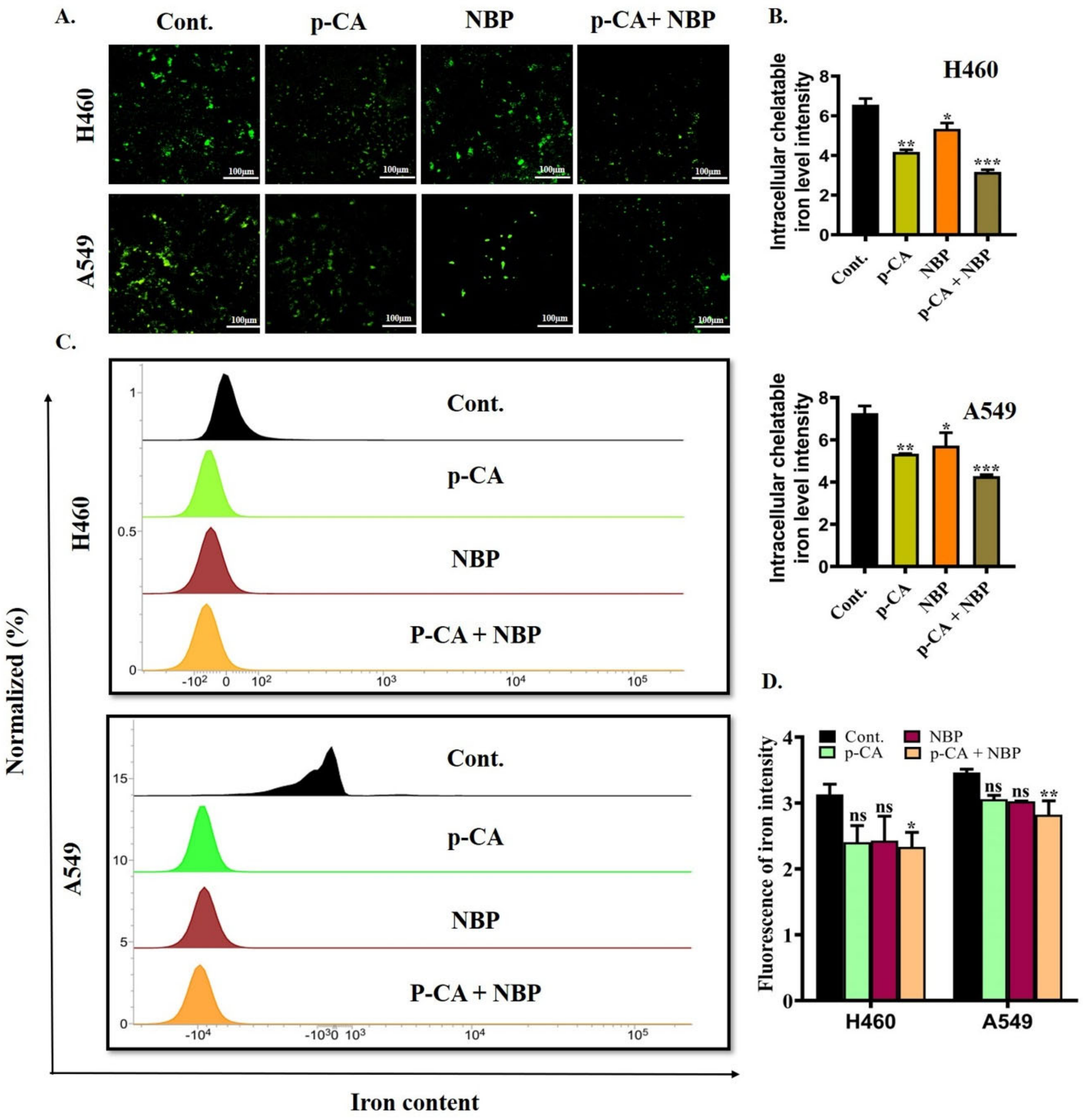

Figure S8. (A) Intracellular chelatable iron level in H460, and A549 cells after 24 hours of para coumaric acid and NBP-jet plasma treatment; (B) the quantitative analysis of chelatable iron level in both cell lines by using ImageJ. (C) The chelatable iron level of H460 and A549 cells by using flow cytometry analysis with Phen Green SK for detecting iron level kit. (D) Quantification of flow cytometry intensity in both cell lines using ImageJ software. The data are presented as the means ± standard deviations of three independent experiments. Significance levels are denoted as *P < 0.05, **P < 0.01, and ***P < 0. 001 vs the control/treated group. ns, not significant.

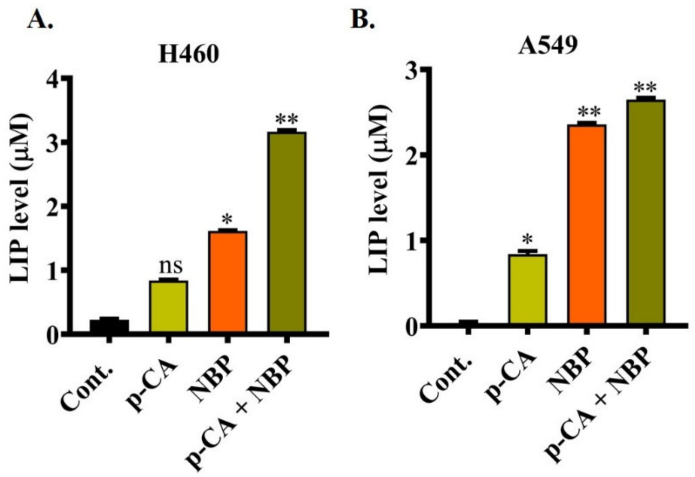

Figure S9. Labile iron pool (LIP) level in (A) H460, and (B) A549 cells after 24 hours of para coumaric acid and NBP-jet plasma treatment. Following treatment, cells were incubated with 5 µM calcein-AM for 30 minutes, and fluorescence emission at 505 nm was subsequently measured. The data are presented as the means ± standard deviations of three independent experiments. Significance levels are denoted as *P < 0.05, **P < 0.01, and ***P < 0. 001 vs the control/treated group. ns, not significant.

Reference

1. Zhu, X.-M.; Pu, Y.-K. Determining the Electron Temperature in Inductively Coupled Nitrogen Plasmas by Optical Emission Spectroscopy with Molecular Kinetic Effects. *Phys. Plasmas* **2005**, *12*.

2. Zhu, X.-M.; Pu, Y.-K. A Simple Collisional--Radiative Model for Low-Temperature Argon Discharges with Pressure Ranging from 1 Pa to Atmospheric Pressure: Kinetics of Paschen 1s and 2p Levels. *J. Phys. D. Appl. Phys.* **2009**, *43*, 15204.

3. Zhu, X.-M.; Pu, Y.-K. Optical Emission Spectroscopy in Low-Temperature Plasmas Containing Argon and Nitrogen: Determination of the Electron Temperature and Density by the Line-Ratio Method. *J. Phys. D. Appl. Phys.* **2010**, *43*, 403001.

4. Choi, E.H.; Kaushik, N.K.; Hong, Y.J.; Lim, J.S.; Choi, J.S.; Han, I. Plasma Bioscience for Medicine, Agriculture and Hygiene Applications. *J. Korean Phys. Soc.* **2022**, *80*, 817–851.

5. Lee, T.; Keidar, M. Adaptive Plasma and Machine Learning. *Plasma Cancer Ther.* **2020**, 223–250.

6. Gu\dhmundsson, J.T. *Electron Excitation Rate Coefficients for the Nitrogen Discharge*; Science Institute, University of Iceland, 2005;

7. Lofthus, A.; Krupenie, P.H. The Spectrum of Molecular Nitrogen. *J. Phys. Chem. Ref. Data* **1977**, *6*, 113–307.
